# Decoding the spatiotemporal regulation of transcription factors during human spinal cord development

**DOI:** 10.1038/s41422-023-00897-x

**Published:** 2024-01-05

**Authors:** Yingchao Shi, Luwei Huang, Hao Dong, Meng Yang, Wenyu Ding, Xiang Zhou, Tian Lu, Zeyuan Liu, Xin Zhou, Mengdi Wang, Bo Zeng, Yinuo Sun, Suijuan Zhong, Bosong Wang, Wei Wang, Chonghai Yin, Xiaoqun Wang, Qian Wu

**Affiliations:** 1 Guangdong Institute of Intelligence Science and Technology, Guangdong, China; 2 State Key Laboratory of Brain and Cognitive Science, Institute of Biophysics, Chinese Academy of Sciences, Beijing, China; 3 University of Chinese Academy of Sciences, Beijing, China; 4 Changping Laboratory, Beijing, China; 5 State Key Laboratory of Cognitive Neuroscience and Learning, IDG/McGovern Institute for Brain Research, New Cornerstone Science Laboratory, Beijing Normal University, Beijing, China

**Keywords:** Neural stem cells, Pluripotency

## Abstract

The spinal cord is a crucial component of the central nervous system that facilitates sensory processing and motor performance. Despite its importance, the spatiotemporal codes underlying human spinal cord development have remained elusive. In this study, we have introduced an image-based single-cell transcription factor (TF) expression decoding spatial transcriptome method (TF-seqFISH) to investigate the spatial expression and regulation of TFs during human spinal cord development. By combining spatial transcriptomic data from TF-seqFISH and single-cell RNA-sequencing data, we uncovered the spatial distribution of neural progenitor cells characterized by combinatorial TFs along the dorsoventral axis, as well as the molecular and spatial features governing neuronal generation, migration, and differentiation along the mediolateral axis. Notably, we observed a sandwich-like organization of excitatory and inhibitory interneurons transiently appearing in the dorsal horns of the developing human spinal cord. In addition, we integrated data from 10× Visium to identify early and late waves of neurogenesis in the dorsal horn, revealing the formation of laminas in the dorsal horns. Our study also illuminated the spatial differences and molecular cues underlying motor neuron (MN) diversification, and the enrichment of Amyotrophic Lateral Sclerosis (ALS) risk genes in MNs and microglia. Interestingly, we detected disease-associated microglia (DAM)-like microglia groups in the developing human spinal cord, which are predicted to be vulnerable to ALS and engaged in the TYROBP causal network and response to unfolded proteins. These findings provide spatiotemporal transcriptomic resources on the developing human spinal cord and potential strategies for spinal cord injury repair and ALS treatment.

## Introduction

Cellular diversity in the vertebrate spinal cord is established along three spatial axes of the embryonic body plan: rostral-caudal, dorsal-ventral, and medial-lateral. The dorsoventral axis is dependent on the activities of morphogens, including BMPs, Wnts, and Shh, which induce spatially restricted expression of transcription factors (TFs) in progenitor cells along the axis.^1–5^ Combinatorial TF expression directs the differentiation of progenitor cells towards molecularly and physiologically distinct neuronal subtypes, opposing adjacent transcriptional programs and sharpening boundaries between progenitor zones.^2,6–9^ Spinal cord development is also organized along a medial-lateral axis, with dividing progenitor cells located medially and differentiating progeny migrating laterally. Over time, a medial-lateral restricted cellular organization is established, comprising dividing progenitors, nascent and mature neurons. Cellular spatial variation is also evident along a rostral-caudal axis, as evidenced by the establishment of segmental divergence of motor neurons (MNs) to adapt to the distinctive characteristics of their peripheral targets through opposing gradients of FGF and RA.^10,11^ Spinal cord development is also tightly regulated temporally,^12,13^ ensuring the construction of functionally complex neural circuits. Disruption of this spatiotemporal system can lead to developmental disorders. While extensively studied in model animals, the mechanisms underlying human spinal cord development are not well understood.

Here, we present a comprehensive spatiotemporal transcriptomic analysis of the developing human spinal cord, spanning from the early first to the early third trimester, utilizing single-cell RNA-sequencing (scRNA-seq) and spatial transcriptome sequencing techniques, including TF-seqFISH and 10× Visium. Our study sheds light on the transcriptional TF regulation during human spinal cord development, demonstrating the efficacy of TF-seqFISH for probing spatial expression of TFs. Our dataset provides a detailed view of the early neurogenic and gliogenic events in the developing human spinal cord, which occur earlier than in the human brain. Furthermore, we uncovered the spatiotemporal organization of transcriptionally heterogeneous progenitor cells along the dorsoventral axis and the orderly arrangement of heterogeneous cells at distinct differentiation status along the medial-lateral axis. We also elucidated the early and late waves of neurogenesis in the dorsal horn of the developing human spinal cord that underlie the expansion and laminar formation of the dorsal horn, identifying gestational week (GW)8 as a critical period of dorsal horn development. A sandwich-like arrangement of excitatory interneurons (Ex-INs) and inhibitory interneurons (In-INs), representing an intermediate state of dorsal horn development shaped by spatiotemporally characteristic neurogenesis and neuron migration, is also transiently observed at GW8. We further delved into MN development and analyzed the genetic programs responsible for MN diversification in the developing human spinal cord. Additionally, we performed an integrated analysis of scRNA-seq and Genome-Wide Association Study (GWAS) datasets, identifying one group of disease-associated microglia (DAM)-like microglia in the developing human spinal cord. By analyzing these DAM-like microglia, we shed light on the potential engagement of microglial cells in the pathogenesis of Amyotrophic Lateral Sclerosis (ALS), a neurodegenerative disease characterized by progressive loss of MNs. Thus, our findings provide a promising alternative for the treatment of this debilitating disease by focusing on microglia. Overall, our study provides important insights into the spatiotemporal transcriptomic landscape of human spinal cord development and the underlying molecular mechanisms.

## Results

### A spatiotemporal transcriptomic atlas of developing human spinal cord

**Fig. 1:**
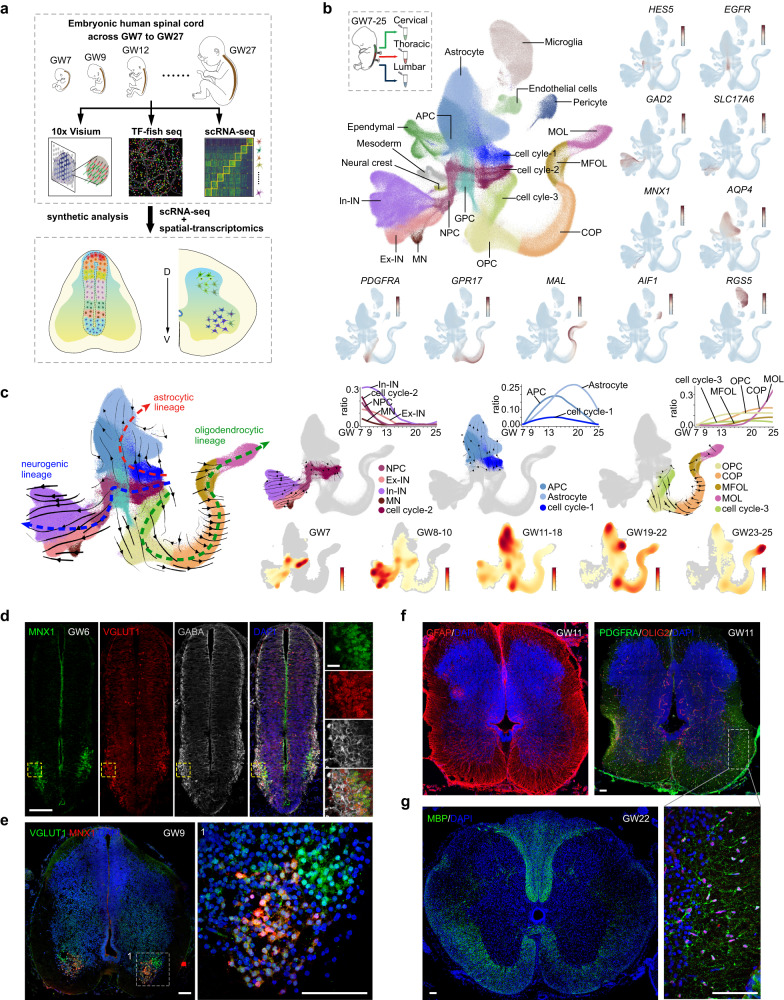
A comprehensive spatiotemporal view of transcriptomic changes during human spinal cord development. **a** A comprehensive spatiotemporally resolved transcriptomic landscape of the developing human spinal cord across GW7–27 was obtained through the use of scRNA-seq, TF-seqFISH, and 10× Visium technologies. This schematic provides an overview of the experimental design and analysis. **b** The developing human spinal cord from GW7–25 was segmented into cervical, thoracic, and lumbar regions and then analyzed using scRNA-seq. Through unsupervised clustering and marker gene expression, 20 distinct cell types were identified and annotated. The expression profiles of marker genes are depicted through a visualization where each dot represents an individual cell colored according to the expression level (dark red, high expression; light blue, low). **c** We present an analysis of the neurogenic, astrocytic, and oligodendrocytic lineages in the developing human spinal cord using RNA velocity (left). To aid interpretation, we have highlighted the three lineage trajectories separately (right). The changes in cell ratio over GWs are depicted by fitted curves, while a density map shows cell density at different gestational stages, with high density denoted by dark red. Unrelated cells were excluded from this UMAP analysis. **d** At GW6, the MNX1^+^ MNs, VGLUT1^+^ Ex-INs, and GABA^+^ In-INs could be easily identified in the ventral region of the spinal cord. The regions of interest are magnified in the boxes. Scale bars,100 μm (left) and 20 μm (right). **e** The spatial separation of MNX1^+^ MNs and VGLUT1^+^ Ex-INs in the human spinal cord at GW9 is demonstrated in this image. The areas within the boxes are shown in higher magnification to provide a better visualization. Scale bars, 100 μm (left), 20 μm (right). **f** The expression patterns of GFAP, OLIG2, and PDGFRA in the human spinal cord at GW11 are presented. The boxed regions are displayed at high magnification. Scale bar, 100 μm. **g** Expression of MBP in human spinal cord samples at GW22 is shown. Scale bars, 100 μm.

We present here a comprehensive spatiotemporal cellular atlas of the developing human spinal cord, based on two sources of transcriptomic data at single-cell resolution. The first dataset was obtained by performing scRNA-seq on individual cells in the developing human spinal cord, covering the early first to early third trimester (GW7–25), resulting in a transcriptional profile of 217,636 single cells. The second dataset consists of two sets of spatially resolved transcriptomic data, obtained using the 10× Visium and a single-cell transcriptomics method that quantified the expression levels of 1085 TFs (TF-seqFISH, see Materials and Methods), respectively (Fig. 1a). To create a more comprehensive cellular landscape, we integrated our scRNA-seq data with data from a previous study,^14^ resulting in a transcriptional census of 912,514 single cells across GW7 to GW25 (Fig. 1b; Supplementary information, Fig. S1a). Twenty major cell clusters were identified through unsupervised clustering and visualized via Uniform Manifold Approximation and Projection (UMAP), which we annotated using well-known cell type-specific markers (Fig. 1b; Supplementary information, Fig. S1b and Tables S1, S2). Our analysis revealed the existence of three cell types in the cell division cycle, labeled as cell cycle-1, -2, and -3, as well as neural progenitor cells (NPCs) and the neuronal types of In-INs, Ex-INs, and MNs (Fig. 1b). In addition, we detected distinct cell types in the oligodendrocyte lineage, including oligodendrocyte progenitor cells (OPCs), committed oligodendrocyte progenitors (COPs), myelin-forming oligodendrocytes (MFOLs), and mature oligodendrocytes (MOLs) (Fig. 1b). We also identified astrocytes and astrocyte progenitor cells (APCs), as well as one group of glial progenitor cells (GPCs) situated between the astrocyte and oligodendrocyte lineages in this UMAP embedding (Fig. 1b). Gene expression analysis revealed that, in addition to markers like *HOPX* and *EGFR*, GPCs displayed expression profiles of both astrocytic (*GFAP*, *AQP4*, and *SLC1A3*) and oligodendrocytic lineages (*OLIG2* and *OLIG2*) (Supplementary information, Fig. S1c). Trajectory analysis further supported the potential bipotent nature of GPCs (Supplementary information, Fig. S1c). Additionally, we discerned microglia, endothelial cells, pericytes, neural crest cells, mesoderm, and ependymal cells (Fig. 1b).

Next, we employed RNA velocity analysis to infer the differentiation lineages leading to neuronal, astrocytic, and oligodendrocytic fates (Fig. 1c). Intriguingly, the differentiation trajectories revealed that the three cell groups in cell division (cell cycle-1/2/3) are involved in astrocytic, neuronal, and oligodendrocytic lineages, respectively, with distinct differentially expressed genes (DEGs) (Fig. 1c; Supplementary information, Fig. S1d and Table S2). These findings suggested that the genetic cues instructing the neurogenic, astrocytic, or oligodendrocytic fates emerge early, even in dividing cells. To elucidate the timing of neurogenic and gliogenic events in the developing human spinal cord, we analyzed the cell ratio dynamics for cells implicated in the neuronal, astrocytic, and oligodendrocytic lineages across GWs (Fig. 1c; Supplementary information, Fig. S1e, f). Our results demonstrate that neurogenesis in the developing human spinal cord is initiated very early, as neurogenic dividing cells (cell cycle-2) and NPCs are present in high proportion at GW7, the earliest stage included in our dataset (Fig. 1c; Supplementary information, Fig. S1e, f). Accordingly, abundant neuronal progenies, including MNs, Ex-INs, and In-INs classified according to their neurotransmitter properties, are detectable as early as GW7 (Fig. 1c; Supplementary information, Fig. S1e, f).

In contrast, gliogenic events are more hysteretic. In the astrocytic lineage, astrocytic dividing cells and APCs are detectable around GW10 and peak at GW15, while astrocytes increase gradually around GW10 and peak at GW20 (Fig. 1c; Supplementary information, Fig. S1e, f). In the oligodendrocyte lineage, the cell types at low differentiation states, such as the oligodendrocytic dividing cells (cell cycle-3), OPCs, and COPs, emerge around GW10 and gradually increase thereafter (Fig. 1c; Supplementary information, Fig. S1e, f). Well-differentiated MFOLs and MOLs arise from GW18 and increase substantially after GW20 (Fig. 1c; Supplementary information, Fig. S1e, f). These temporal rules of neurogenic and gliogenic events in the developing human spinal cord are further supported by immunofluorescence staining, where the MNX1^+^ MNs, VGLUT1^+^ Ex-INs, and GABA^+^ In-INs can be detected as early as GW6, particularly in the ventral horn, indicating that neurogenesis in the human spinal cord may occur even earlier than GW6 (Fig. 1d). Besides, we also probed the spatially characterized organization of MNX1^+^ cells and VGLUT1^+^ cells at GW9 (Fig. 1e). Furthermore, we conducted immunostaining for genes related to glia differentiation in embryonic human spinal cord. Although absent at GW8, abundant GFAP+ astrocytes and PDGFRA^+^OLIG2^+^ OPCs could be detected at GW11 (Fig. 1f; Supplementary information, Fig. S1g). Additionally, at GW22, MBP^+^ oligodendrocytes were detected in the white matter of the spinal cord (Fig. 1g; Supplementary information, Fig. S1g). Taken together, our results demonstrate the early initiation of neurogenesis in the human spinal cord around GW6 and the hysteretic gliogenesis occurring around GW10. Compared with the counterpart events in the developing human cerebral cortex,^15–18^ the early initiation of neurogenesis and gliogenesis, especially the gliogenic events, is evident in the developing human spinal cord (Supplementary information, Fig. S1h).

### Unraveling the spatial organization of neural progenitor cells with TF-seqFISH

**Fig. 2:**
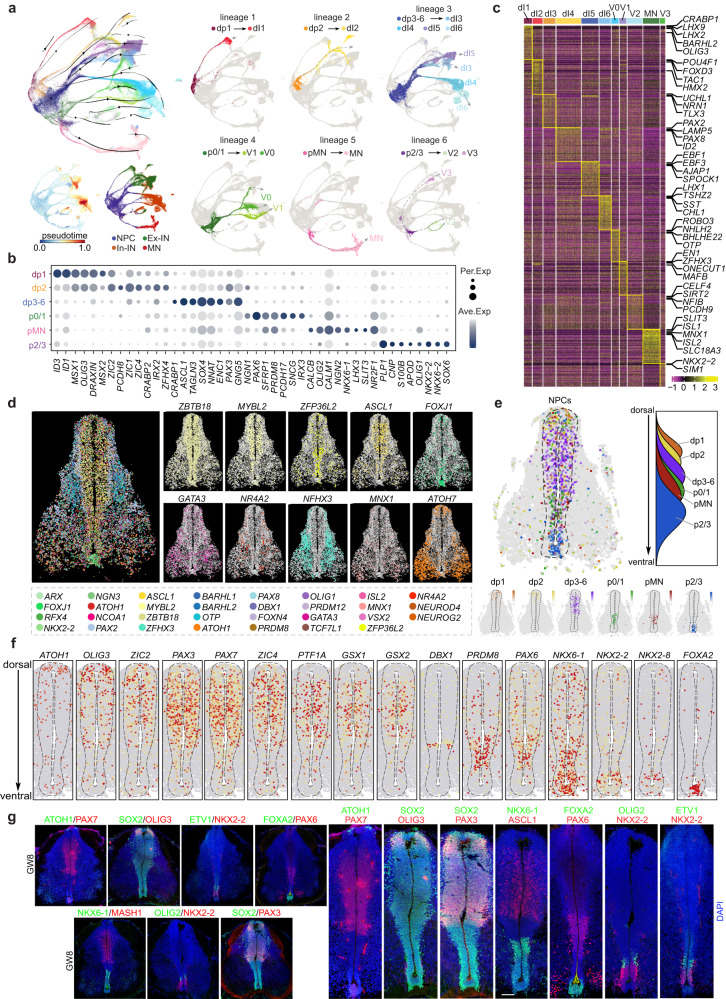
Molecular and spatial characterization of neural progenitor cells in the developing human spinal cord. **a** The distinct neuronal lineages comprising NPCs and neurons are visualized using a UMAP plot that integrates the outcomes of RNA velocity analysis. To enhance clarity, each neuronal lineage is also depicted individually, and illustrated with gray dotted lines. The cells are colored based on their identities as NPCs, Ex-INs, In-INs, and MNs, their pseudotime scores, or based on their identities in different lineages. **b** The expression profiles of DEGs across diverse NPC subtypes are shown in a dotplot. **c** A heatmap depicting the expression patterns of DEGs enriched in various neuronal types in the developing human spinal cord is presented. **d** RNA signals for 31 highly variable genes on a coronal section of the human spinal cord at GW8 are spatially presented, with each dot representing a single molecule of RNA. The spatial expression profiles of some example genes are shown individually for better visualization. **e** The spatiotemporal arrangement of NPCs along the dorsoventral axis in the VZ of human spinal cord is inferred through an integrated analysis of scRNA-seq and TF-seqFISH data. The spatial distribution of NPC cell types is represented as a simplified density map based on the dorsoventral score. The NPCs are arranged along the DV axis from dorsal to ventral as: dp1, dp2, dp3–6, p0–2, pMN, and p3. **f** The spatial expression patterns of TFs in the VZ along the dorsoventral axis of the human spinal cord at GW8 are visualized. Each dot represents an individual cell and is colored according to the expression level of the TF (red, high; gray, low). The dotted lines indicate the boundaries of the VZ in the human spinal cord at GW8. **g** The immunostaining results visualize the spatial expression patterns of TFs, including OLIG3, PAX3, NKX6-1, ASCL1, FOXA2, OLIG2, and NKX2-2, in the VZ of the human spinal cord at GW8. Scale bar, 50 μm.

To investigate the molecular characteristics and spatial organization principles of neurogenesis in the embryonic human spinal cord, we conducted scRNA-seq analysis on extracted NPCs and neurons. Through the implementation of unsupervised clustering, RNA velocity analysis and subsequent visualization using UMAP, we identified distinct neuronal lineages that exhibited unique molecular signatures (Fig. 2a; Supplementary information, Fig. S2a). The NPCs within each lineage were found to have low pseudotime values and served as initiating cells (Fig. 2a). We annotated these lineages based on characteristic expression of accredited TF markers, defining distinct NPC pools of dp1, dp2, dp3–6, p0, p0/1, pMN, and p2/3, and corresponding neuronal types of dI1, dI2, dI3, dI4, dI5, dI6, V0, V1, MN, V2, and V3 (Fig. 2a–c; Supplementary information, Fig. S2a). For instance, mouse TF markers for dp1 (e.g. *MSX1*, *MSX2*) and dI1 (e.g. *LHX2*, *LHX9*, and *BARHL1*) were found to be specifically enriched in human NPCs and neurons in lineage 1, which we therefore defined as dp1 and dI1, respectively (Fig. 2a–c; Supplementary information, Fig. S2a). We identified six neuronal lineages in the developing human spinal cord, each with a characteristic cell composition. Lineage 1 consisted of dp1 and dI1; lineage 2 depicted the differentiation from dp2 towards dI2; lineage 3 comprised heterogeneous cell types of dp3–6 and dI3, dI4, dI5, dI6; lineage 4 illustrated the bifurcated differentiation of p0–1 towards V1 and V0; lineage 5 demonstrated the specification of pMN to MN; and lineage 6 illustrated the differentiation of p2–3 towards V2 and V3 (Fig. 2a; Supplementary information, Fig. S2a). Notably, certain cell types that are well-distinguished in mice were difficult to discern in the human fetal spinal cord due to the similarity in molecular features, such as the dp3–6, p0–1, and p2–3 (Fig. 2a–c; Supplementary information, Fig. S2a). Besides, we observed that neurons with the same neurotransmitter properties were consistently clustered together, with glutamatergic dI3 and dI5 neurons close together in the UMAP, as were the GABAergic dI4 and dI6 (Fig. 2a; Supplementary information, Fig. S2a). Additionally, we identified novel gene expression patterns beyond well-known genetic markers. For instance, we found specific expression of *CRABP1*, *LHX2* and *BARHL2* in dI1, *EBF1*, *EBF3*, and *AJAP1* in dI5, *THSZ2*, *SST*, and *CHL1* in dI6, as well as *SLIT3*, *ISL1* and *ISL2* in MNs (Fig. 2c). Immunostaining was performed for CRABP1 along with the classical dI1 marker LHX2, as well as for SLIT3 along with the MN-specific CHAT. The co-expression of the newly identified genes alongside well-established cell type markers validates our identification of novel genes characteristic of certain cell types (Supplementary information, Fig. S2b).

TFs are essential in regulating neural progenitor domains, cell specification, and the patterning of brain regions during development.^19,20^ In this study, we introduced an image-based single-cell TF-seqFISH technique to examine the expression profiles of 1085 TFs in the developing human spinal cord, in a spatial context. Our emphasis was on deciphering the spatial distribution of TFs within the ventricular zone (VZ)-resident NPCs. So, we performed a comprehensive analysis to determine the suitable spinal tissues for performing TF-seqFISH. We initially performed immunostaining for SOX2 and NEUN to label NPCs in the developing human spinal cord at various stages. The results demonstrated that SOX2^+^ NPCs were notably abundant at GW8, congregating within a thickened VZ region. However, as development progresses, the SOX2^+^ NPC-enriched VZ layer gradually diminishes (Supplementary information, Fig. S2c). Next, we analyzed RNA levels in the NPCs of spinal cord tissues at different time points, revealing that a higher number of RNA molecules were detected at an early stage, especially at GW7–9. (Supplementary information, Fig. S2d). Furthermore, we also took into account the availability of spinal cord tissues and the temporal alignment with the scRNA-seq dataset. Thus, we conducted TF-seqFISH on human spinal tissues collected at GW8, yielding a cellular-resolution spatial mapping of TF mRNA within the developing spinal cord slices containing 12,274 cells (Supplementary information, Fig. S2e). Our visualization of 31 highly variable genes with spatial expression profiles revealed the heterogeneity of TF mRNA in VZ-restricted NPCs along the dorsoventral axis (Fig. 2d; Supplementary information, Fig. S2e). By integrating scRNA-seq and TF-seqFISH datasets, we were able to assign unique spatial identities to each VZ cell, including dp1, dp2, dp3–6, p0/1, p2/3, and pMN, respectively, along the dorsoventral axis (Supplementary information, Fig. S2f). The spatial assignments of NPCs along the dorsoventral axis (dp1→dp2→dp3–6→p0/1→pMN→p2/3) in our study are similar to those reported in developing mouse and chick spinal cords^7,13^ (Fig. 2e).

Furthermore, we investigated the spatial organization pattern of TF configuration that contributes to progenitor subdivision in humans. Our results showed the spatially restricted expression of TFs that are crucial for defining progenitor identities in the VZ region (Fig. 2f; Supplementary information, Fig. S2g). Specifically, *ATOH1*, which is dp1-specific, is mainly enriched in the dorsal-most cells, while dp3–6 enriched *PAX3*, *PAX7*, *GSX1*, and *GSX2* are widely distributed in the middle-upper part (Fig. 2f; Supplementary information, Fig. S2g). Additionally, we observed the ventrally restricted expression of TFs such as *PRDM8*, *NKX6-1*, *NKX2-2*, *NKX2-8*, and *FOXA2* (Fig. 2f; Supplementary information, Fig. S2g). These findings were validated by immunostaining (Fig. 2g). While our study unveiled the remarkable conservation of spatial gene expression in spinal NPCs across species evolution, we also identified distinct features unique to human spinal cord development. For instance, *PTF1A* is widely distributed in the human spinal cord, with a pattern comparable to that of dp3–6 specific *GSX1*, *GSX2*, *PAX3*, and *PAX7* (Fig. 2f; Supplementary information, Fig. S2g), unlike its restriction to dp4 neurons in the mouse spinal cord.^21^ This difference may explain the indiscernibility of dp3–6 progenitor cells in the human spinal cord.

In summary, our study not only identified transcriptionally diverse NPCs based on scRNA-seq analysis but also elucidated the spatial organization pattern of NPC and TF expression in the developing human spinal cord. Our findings suggested that, although the spatial transcriptome of NPCs was largely conserved in species evolution, human fetal spinal cord development followed subtly different genetic regulatory rules.

### Revealing spatial dynamics of neuronal differentiation and migration along the medial-lateral axis using TF-seqFISH

**Fig. 3:**
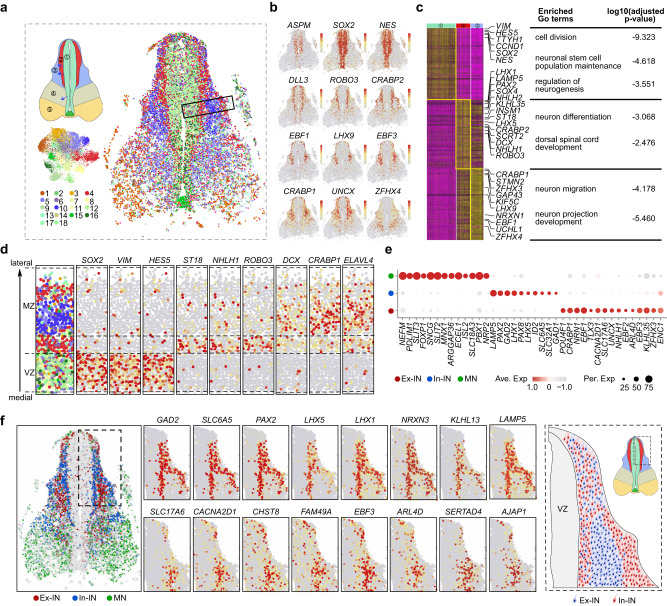
Spatially resolved characterization of the mediolateral organization pattern in the developing human spinal cord. **a** Unsupervised clustering in TF-seqFISH dataset identified 18 molecularly defined clusters and their corresponding spatial localization in a slice of developing human spinal cord at GW8. Individual cells are represented by dots and colored based on the clusters, revealing five distinct anatomical regions based on spatially restricted cell distribution. To aid clarity, a schematic illustration is also provided, where distinct regions are denoted by digits and blue arrows indicate migratory directions of neurons in the spinal cord. **b** Visualization of the imputed gene expression profiles in the developing human spinal cord, with each dot representing an individual cell and color coded according to the expression level (red, high; gray, low). **c** A heatmap depicting the expression profiles of DEGs among cells located in Regions 1, 2, and 3 of the developing human spinal cord is presented, with enriched GO terms for the DEGs listed. **d** Visualization of laminar cell organization and gene expression, including TFs and non-TF genes, along the mediolateral axis in the dorsal horn of the developing human spinal cord facilitated by utilizing the imputed data. Each dot represents an individual cell and is colored based on expression level (red, high; gray, low). The leftmost column is a zoomed-in image of the boxed area in **a**. **e** A dot plot illustrating the expression profiles of DEGs among Ex-INs, In-INs, and MNs in the developing human spinal cord. **f** The spatial organization of Ex-INs, In-INs, and MNs in the developing human spinal cord is displayed, with the spatially-restricted expression of genes specific for Ex-INs and In-INs in the dorsal horn also depicted in the boxed area based on the imputed data. A schematic representation of the sandwich-like arrangement of Ex-INs and In-INs in the dorsal horn is provided for clarity.

Following the delineation of the spatial transcriptome of NPCs, we sought to elucidate the rules governing neuronal organization in the developing human spinal cord. To this end, we employed unsupervised classification based on the expression of 1085 TFs in the TF-seqFISH datasets, which resulted in the identification of 18 distinct molecular clusters (Fig. 3a, b; Supplementary information, Fig. S3a, b). These clusters were further demarcated into 5 spatially discrete domains (Region 1–5) through a correlation-based analysis (Fig. 3a; Supplementary information, Fig. S3b–c). Specifically, Clusters 2, 7, 9, 11, 12, 16, and 18 were associated with Region 1; Clusters 4 and 13 were assigned to Region 2; Cluster 10 was related to Region 3; Clusters 14 and 5 were located in Region 4; Clusters 1, 3, 6, 8, and 17 were grouped in Region 5 (Fig. 3a; Supplementary information, Fig. S3b, c). According to the developing human spinal cord atlas,^22^ Region 1, located medially, primarily consists of dividing progenitor cells. Region 2, an intermediate zone (IZ) adjacent to the proliferative areas, is designated as a Sojourn zone, and contains premigratory neurons. The dorsally localized mantle zone, Region 3, is comprised of migrating and settling dorsal horn neurons. Region 4, an in-between mantle zone (MZ), contains intermediate interneurons and migrating neurons towards the ventral horn. Finally, the ventral horn-specific Region 5 is mainly composed of settled ventral horn neurons (Fig. 3a). These TF-classified spatial territories provide a valuable molecular basis for delineating neuroanatomical structures.

Then, we have imputed expression profiles of transcriptome-wide genes by combining the TF-seqFISH and scRNA-seq data (Supplementary information, Fig. S2f). Leveraging the imputed data, we deduced the spatial expression patterns of non-TF genes as well, providing a comprehensive resource for decoding the cellular and molecular organization principles of the developing human fetal spinal cord. With this dataset, we aimed to unravel the enigma of neuron migration and differentiation in the human spinal cord. In the dorsal horn, Regions 1, 2, and 3 are highly laminated (Fig. 3a). Analysis of DEGs and gene ontology (GO) reveals that VZ cells in Region 1 are involved in cell division, maintenance of the neuronal stem cell population, and regulation of neurogenesis (Fig. 3c; Supplementary information, Table S3). Conversely, cells in the laterally localized Regions 2 (IZ) and 3 (MZ) are implicated in neuron differentiation, migration, projection development, and dorsal spinal cord development (Fig. 3c; Supplementary information, Table S3).

The transition from a proliferative progenitor cell to a post-mitotic neuron is tightly regulated by different molecules. For instance, we found that *SOX2*, *VIM*, and *HES5* are enriched in VZ region (Region 1), whereas *ST18*, *NHLH1*, *ROBO3*, and *INSM1* are spatially restricted to IZ (Region 2). In comparison, *DCX*, *CRABP1*, and *ELAVL4* expressions are confined in the more laterally MZ (Region 3) (Fig. 3c, d; Supplementary information, Table S3). Among the genes enriched in Region 2 cells, *NHLH1* and *INSM1* are transiently expressed in late neuronal progenitors and nascent neurons,^23,24^ further indicating the nascent identity of Region 2 cells that have not yet fully differentiated and reached their final locations. Hence, by probing the specific gene expression in Region2, we identified a group of early markers for post-mitotic neurons in the IZ of the developing human spinal cord, including *NHLH1*, *INSM1*, *ST18*, *ROBO3*, *KLHL35*, and more. Additionally, we identified genes expressed in later, more mature, post-mitotic cells in the lateral MZ (Region 3), such as *CRABP1*, *STMN2*, *DCX*, and *ZFHX3* (Fig. 3c, d). Similar results were achieved in the ventral part of the developing spinal cord (Supplementary information, Fig. S3d, e and Table S4). However, the ventral proliferative VZ region is much thinner compared to its dorsal counterpart, potentially due to earlier neurogenesis in the ventral region (Fig. 3a; Supplementary information, Fig. S3e).

Overall, our TF-seqFISH dataset provided an invaluable resource for understanding the cellular and molecular basis underlying the neuroanatomical demarcation in the developing human spinal cord. Furthermore, our dataset provided a detailed understanding of the spatial and molecular programs underlying neuron generation, migration, and differentiation.

### Spinal dorsal horn development in the developing human spinal cord

The spinal cord neurons can be further categorized based on their neurotransmitter properties, which play crucial roles in neuronal function and circuit connectivity. There are three main types of neurotransmitter-defined neurons in the spinal cord: glutamatergic Ex-INs, GABAergic/glycinergic In-INs, and acetylcholinergic MNs. Through DEG analysis of the scRNA-seq dataset, we identified characteristic molecular profiles that distinguish these neuronal types, such as *NEFM*, *SLIT2*, *SNCG*, *ECEL1*, and *NRP2* in MNs, *LAMP5*, *PAX2*, *PAX8*, and *ID2* in In-IN, and *TLX3*, *NRN1*, *EBF3*, *CACNA2D1*, and *CRABP1* in Ex-IN (Fig. 3e; Supplementary information, Table S5). To further investigate the spatial distribution of these three neuronal types, we mapped the TF-seqFISH cells to the transcriptomic taxonomy and assigned the best-matched neuronal identities. The results showed that MNs exclusively reside in the ventral horn, while Ex-IN and In-IN are mainly distributed in the dorsal horn and the intermediate part (Fig. 3f). Interestingly, at GW8, the Ex-INs are sandwiched between two spatially bifurcated groups of In-INs in the dorsal horn (Fig. 3f). Notably, the In-INs located in close proximity to VZ show an intermingling pattern with some Ex-INs (Fig. 3f). The spatial expression profiles of Ex-INs and In-INs enriched genes also reveal this distinctive organization pattern, which remains consistent across the entire spinal cord (Fig. 3f; Supplementary information, Fig. S3f). In the adult spinal cord, dorsal horn neurons are organized into distinct laminae that receive specific sensory inputs.^25^ The sandwich-like organization of Ex-INs and In-INs in the dorsal horn of the human fetal spinal cord may be an intermediate state for the formation of appropriate circuit connections for perceiving environmental and internal signals.

**Fig. 4:**
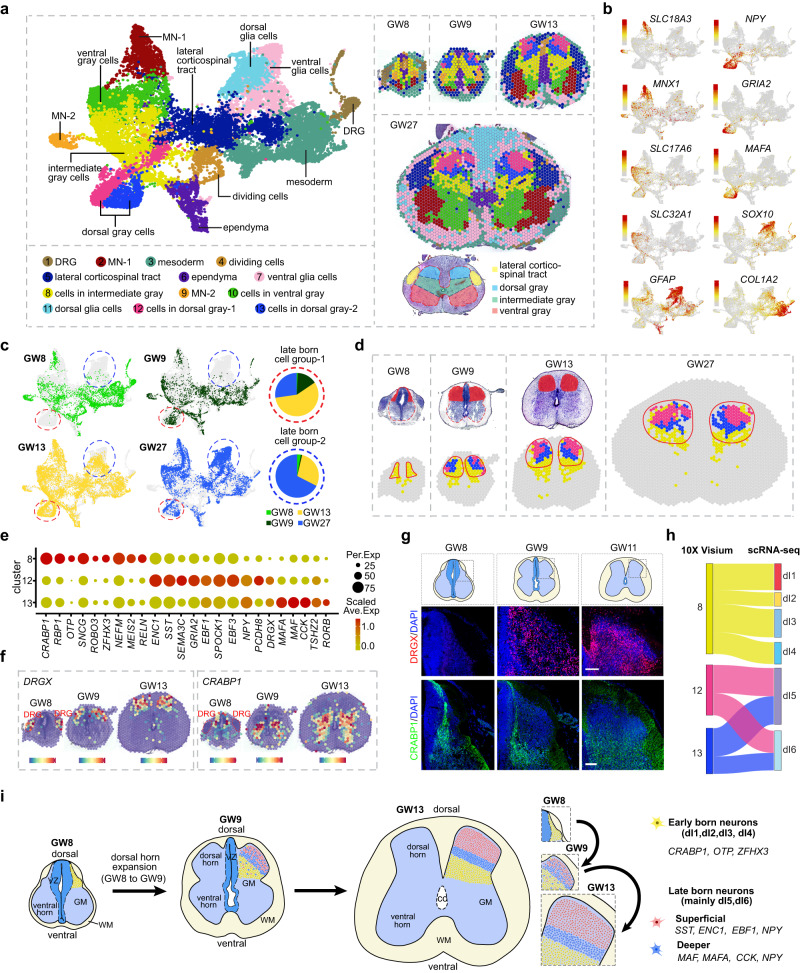
Exploring the spatial, cellular, and molecular bases of dorsal horn development in the human spinal cord. **a** A molecular-based classification of cells in the developing human spinal cord at various stages was performed using the dataset of 10× Visium. Unsupervised clustering was used to identify spots with a shared molecular profile, which were then annotated according to gene expression and spatial position (left). The spatial localization of these molecularly-defined cell groups was visualized in tissue slices at distinct timepoints (right). **b** The UMAP visualization displays the expression profiles of marker genes utilized to annotate spot clusters. Each dot represents an individual spot, colored based on the expression level (red, high; gray, low). **c** Distinct groups of spots derived from different embryonic stages were displayed separately. Notably, a group of spots primarily composed of Clusters 12 and 13, which were absent at GW8, were outlined with red circles. Furthermore, a group of spots mainly composed of Clusters 7 and 11, which were not detected at GW8 and GW9, were also identified and marked with blue circles. **d** The H&E staining images, with red insets highlighting the dorsal horns of the spinal cord at GW8 and GW9, reveal a marked expansion of the dorsal horn from GW8 to GW9. Notably, only spots in cluster 8 are detectable in the dorsal horn at GW8, whereas at GW9, spots from Clusters 12 and 13 emerge and are situated in a superficial layer of the dorsal horn. **e** A dotplot was generated to display the expression profiles of DEGs among Clusters 8, 12, and 13. The size of each dot in the plot is related to the detection rate, while the color bar is scaled with the average gene expression levels. **f** Spatially-resolved transcriptomic expression features of Cluster 8-specific *CRABP1*, as well as Clusters 12 and 13-specific *DRGX* at different developmental stages, are visualized on the 10× Visium platform. These features are overlaid with H&E staining to illustrate the anatomical structures. The DRG regions are outlined. **g** Immunostaining was performed on human spinal cord slices at GW8, 9, and 11 to examine the spatial expression features of DRGX and CRABP1. Scale bars, 100 μm. **h** The neuronal identities of Clusters 8, 12, and 13 have been determined through an integrated analysis of the datasets from 10× Visium and scRNA-seq, and are depicted in a Sankey diagram. **i** Schematic depiction of the cellular rules underlying the expansion and lamina formation of the dorsal horn during human spinal cord development.

Next, we aimed to uncover the molecular and cellular events underlying the development of the dorsal horn over a broad time range. To this end, we enriched our resources of spatial transcriptome data by conducting 10× Visium experiments on developing human spinal cord tissues at various gestational ages (GW8, GW9, GW13, and GW27). Using unsupervised clustering, we categorized 13 molecularly distinctive spot clusters based on molecular features and spatial location, including ventral gray cells, dorsal gray cells, intermediate gray cells, lateral corticospinal tract, dividing cells, ependyma, mesoderm, dorsal root ganglion (DRG) cells (merely captured in the tissues from GW8 and GW9), dorsal and ventral glial cells, and two groups of MNs (Fig. 4a, b; Supplementary information, Fig. S4a–d and Table S6). We found that a group of spots, mainly comprising Clusters 12 and 13 (outlined by red circles), was absent in the GW8 samples (Fig. 4c), and this group of spots was located superficially in the dorsal horn when spatially visualized (Fig. 4d). At GW9, GW13, and GW27, in addition to some yellow spots with identities of Cluster 8, we could also detect the spots of Clusters 12 and 13 in the dorsal horn (Fig. 4d). However, at GW8, only spots of Cluster 8 could be detected in the dorsal horn while those of Clusters 12 and 13 were entirely missing (Fig. 4c, d). Our results indicated that there were early-born neurons that emerged at or before GW8 (Cluster 8) as well as late-born neurons that arose after GW8 (Clusters 12 and 13). The emergence of late-born neurons at GW9 coincides with the expansion of the dorsal horn in the developing human spinal cord, indicating their importance in dorsal horn construction (Fig. 4d).

Moreover, we performed a DEG analysis to probe the molecular characteristics of early- and late-born neurons in the dorsal horn. We found that the early-born neurons in Cluster 8 were specific for *CRABP1*, *RBP1*, *ROBO3*, *MEIS2*, and *OTP*, whereas the late-born neurons in clusters 12 and 13 exhibited a distinctive molecular specificity (Fig. 4e). Cluster 12 was distinctive for *ENC1*, *SST*, *EBF3*, *GRIA2*, and *PCDH8*, while Cluster 13 was characteristic for *MAF*, *MAFA*, *CCK*, and *RORB* (Fig. 4e). The specific enrichment of *CRABP1* in Cluster 8 was also established by analyzing its expression across all distinct clusters (Supplementary information, Fig. S4e). The modular architecture of the dorsal horn was further illustrated by the spatial expression profiles of *CRABP1* and *DRGX*, which symbolize early- and late-born neurons, respectively (Fig. 4f, g). Notably, DRGX exhibited enriched expression specifically in the DRG cells but not in the spinal cord at GW8 (Fig. 4f). We also identified the neuronal identities of Clusters 8, 12, and 13 by integrating scRNA-seq and 10× Visium datasets. The early-born neurons in Cluster 8 were mapped to the neuronal types of dI1, dI2, dI3, and dI4, while those of late-born Clusters 12 and 13 were mainly assigned to dI5 and dI6 (Fig. 4h). Furthermore, our analysis of the scRNA-seq dataset for GW8 and GW9 revealed that the majority of dI5 and dI6 neurons originate from GW9, suggesting that these neurons possess a late-born identity (Supplementary information, Fig. S4f). Intriguingly, aligning with the observations delineated in Fig. 1c–g, our investigation also revealed the tardy emergence of dorsal and ventral glial cells (Fig. 4c; Supplementary information, Fig. S4g).

Thus, by adopting a spatial perspective, we have conducted a comprehensive inquiry into the cellular and molecular mechanisms that underpin the development of the dorsal horn in the human spinal cord (Fig. 4i). During the initial embryonic stages, we have unearthed the prototypical dorsoventral laminar configuration of neurons in the dorsal horn, which lays the groundwork for the constitution of somatosensory circuits. Employing a spatial transcriptomic dataset, we have not only identified early neurogenic events, but also the delayed ones in the dorsal horn of the developing human spinal cord, with the epoch of GW8 ascribing a pivotal temporal interval for the ontogeny of the dorsal horn.

### Molecular and spatial diversification of human MNs throughout spinal cord development

**Fig. 5:**
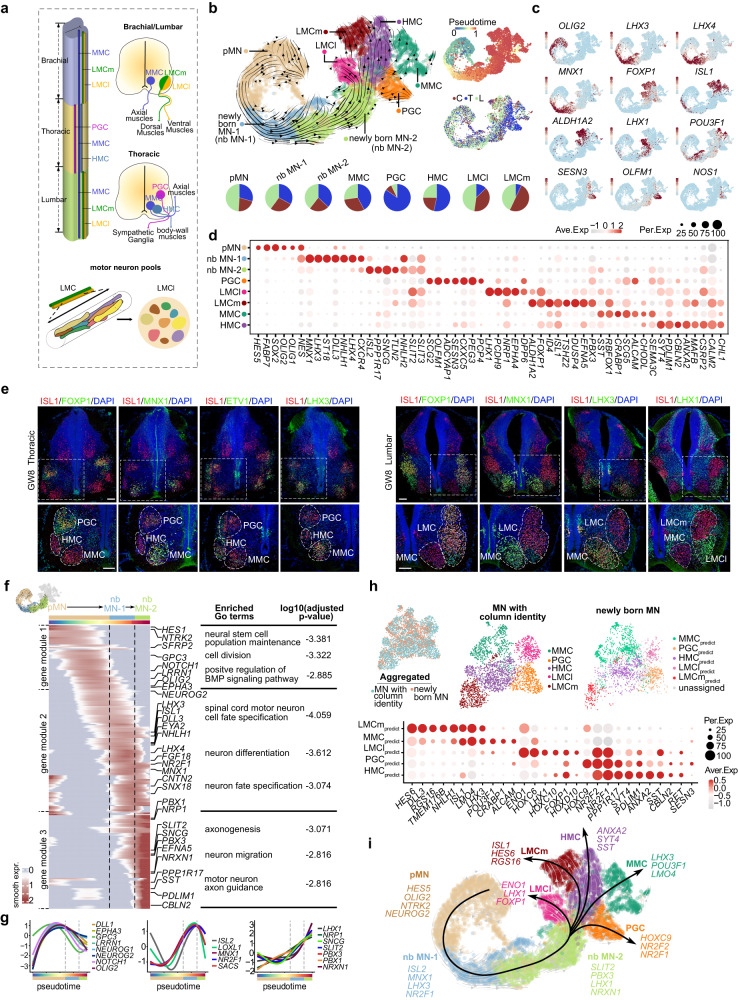
The molecular programs account for the early specification of MN diversity. **a** Schematic illustration to visually depict the organization of MN columns and pools across distinct spinal segments. **b** The ontogeny of MNs is deduced through RNA velocity analysis, which identifies the lineages of pMN, nascent MNs (newly born MN-1 and -2), and MNs with diverse columnar identities (LMCl, LMCm, HMC, PGC, and MMC) based on unique gene expression signatures, visualized through a UMAP plot. The analysis also presents pseudotime and segmental information, and the segmental cell composition of each MN subtype is represented by a pie chart. **c** The expression profiles of hallmark marker genes are depicted using a UMAP visualization. Each dot represents an individual cell and is color-coded according to its expression level (red, high; gray, low). **d** A dotplot visualizing the expression profiles of DEGs across various cell types of MNs is presented. **e** The identities of MNs in the developing human spinal cord have been accurately delineated at GW8, as evidenced by robust immunostaining in the thoracic and lumbar segments. Scale bars, 100 μm. **f** A developmental trajectory from pMN to newly born MN-2 has been identified using RNA velocity analysis, and a heatmap displaying the dynamic gene expression changes along pseudotime is presented. Furthermore, the corresponding enriched GO terms are also listed. **g** A depiction of the sequential waves of gene expression with similar patterns along the pseudotime axis is presented. **h** Upper: The aggregated dataset of newly born MNs and MNs with column identities is depicted using UMAPs, as well as the individual datasets of MNs with distinct column identities and newly born MNs with assigned column identities. Lower: The differential gene expression profile among newly born MNs with predicted fates is presented via a dotplot. **i** Schematic illustration of the genetic programs underlying MN differentiation and diversification.

Spinal motor neurons exhibit diversity in their morphology, connectivity, and functional characteristics.^26,27^ A notable aspect of the MN diversity is their categorization into distinct columnar groups, each of which occupies a specific rostrocaudal position within the spinal cord and innervates a unique array of peripheral target tissues. There are four key categories of MN columnar groups: LMC (Lateral Motor Column), which can be further divided into lateral (LMCl) and medial (LMCm) subcolumns; PGC (Visceral Preganglionic Column); HMC (Hypaxial Motor Column) and MMC (Median Motor Column) (Fig. 5a).

After elucidating the intricate cellular and molecular principles that govern neuronal circuit formation in the dorsal horn, we directed our efforts towards unraveling the factors that drive MN diversification, the sole means of responding to the perceived peripheral cues. To achieve this goal, we delved into the scRNA-seq datasets comprising MNs and their progenitors, which were further categorized into progenitor cells of MN (pMNs), newly born MNs (nb MN-1 and nb MN-2), and MNs with distinct column identities (LMCm, LMCl, MMC, PGC, and HMC), based on unsupervised clustering and remarkable gene expression patterns (Fig. 5b–d; Supplementary information, Table S7). The developmental trajectory was inferred using RNA velocity, which revealed the differentiation of MNs from pMN through newly born MN to MNs with distinct column identities (Fig. 5b). Segmental preference for each cell type, i.e., cervical, thoracic, and lumbar derivation, was also determined (Fig. 5b). Intriguingly, our analysis revealed the early specification of columnar identity of MNs at GW7–8, the earliest time point included in our study (Fig. 5b–d). Furthermore, we validated this early specification of columnar identities, along with their segmental preference, using immunostaining (Fig. 5e). Our study also uncovered novel column-specific gene expression patterns, adding a new dimension to our understanding of the column-distinctive MNs. For instance, we discovered the enrichment of *TAC1*, *NRP1*, and *NTRK2* in LMC, the specification of *TSHZ2*, *DUSP4*, and *ID4* in LMCm, *PCDH9*, *DPP6*, and *OPRK1* in LMCl, as well as the upregulation of *CXXC5*, *PEG3*, and *CELF4* in PGCs (Fig. 5d; Supplementary information, Fig. S5a).

The distinct TF expression profiles are associated with MN pools destined for different target muscle groups.^28^ We identified subgroups within LMC neurons with distinctive transcriptomic signatures. These subgroups, referred to as putative motor neuron pools, were discerned by assessing the distinctive expression of pool-specific genes, such as *NKX6-1*, *NKX6-2, ETV1* and *ETV4* (Supplementary information, Fig. S5b and Table S8). In addition, we also revealed the transcriptional distinction between visceral MNs (mainly the sympathetic PGCs with thoracic derivation) and somatic MNs (including LMCm, LMCl, MMC, and HMC), which was validated by the spatial transcriptome of cervical and thoracic spinal slices at GW13 (Supplementary information, Fig. S5c, d). Thus, our analysis revealed the early transcriptional specification of putative MN columns and MN pools, which is crucial for directing axonal trajectory and nerve-muscle connectivity during development. However, within our datasets, the somatic MNs do not exhibit clear subclustering into alpha and gamma subtypes.^27^ This observation may be attributed to the feature of our dataset, as it predominantly includes MNs derived from early embryonic stages.

To investigate the molecular program driving MN differentiation, we first analyzed the DEGs among cervical, thoracic, and lumbar segments, especially the rostrocaudal HOX expression, which is believed to direct segmentally distinct MN column and pool identities (Supplementary information, Fig. S5e). Next, leveraging developmental trajectory inference, we uncovered the molecular cascade underlying pMN differentiation, which is partitioned into three distinct gene modules enriched in pMN, nbMN-1, and nbMN-2, respectively (Fig. 5f; Supplementary information, Fig. S5f). Functional inference revealed that gene module 1, containing genes such as *DLL1*, *EPHA3*, *OLIG2*, *NEUROG1*, *NEUROG2*, and *GPC3*, is involved in maintaining and dividing neural stem cell populations. Gene module 2, consisting of genes such as *LHX3*, *ISL1*, *MNX1*, *NR2F1*, and *CNTN2*, is involved in specifying spinal cord MN cell fates and promoting neuron differentiation. Finally, gene module 3, comprising genes such as *SLIT2*, *LHX1*, *PBX1*, *PBX3*, and *NRXN1*, is related to axogenesis, MN axon guidance, and neuron migration (Fig. 5f, g; Supplementary information, Fig. S5f). The spatial distribution of these pMN, newly born MN-1 and newly born MN-2 enriched genes along a mediolateral axis is demonstrated based on our TF-seqFISH data (Supplementary information, Fig. S5g). Furthermore, by performing pairwise integration of newly born MNs with MNs in distinct columns, we identified the newly born MNs with putative MMC, PGC, HMC, LMCl, and LMCm fates, thus uncovering the column-specific transcriptional specification in the newly born MNs (Fig. 5h). This is further supported by the robust parallels between the unsupervised subclusters of newly born MNs and the column-specific transcriptional signatures (Supplementary information, Fig. S5h).

Overall, our study reveals the early transcriptomic specification of MNs in organizational (MN columns and pools) and functional (somatic MNs and visceral MNs) contexts. Furthermore, our findings provide a roadmap for programming stem cells into specific MN subtypes by uncovering the transcriptional programs that govern MN diversification (Fig. 5i).

### Astrogenesis during human spinal cord development

**Fig. 6:**
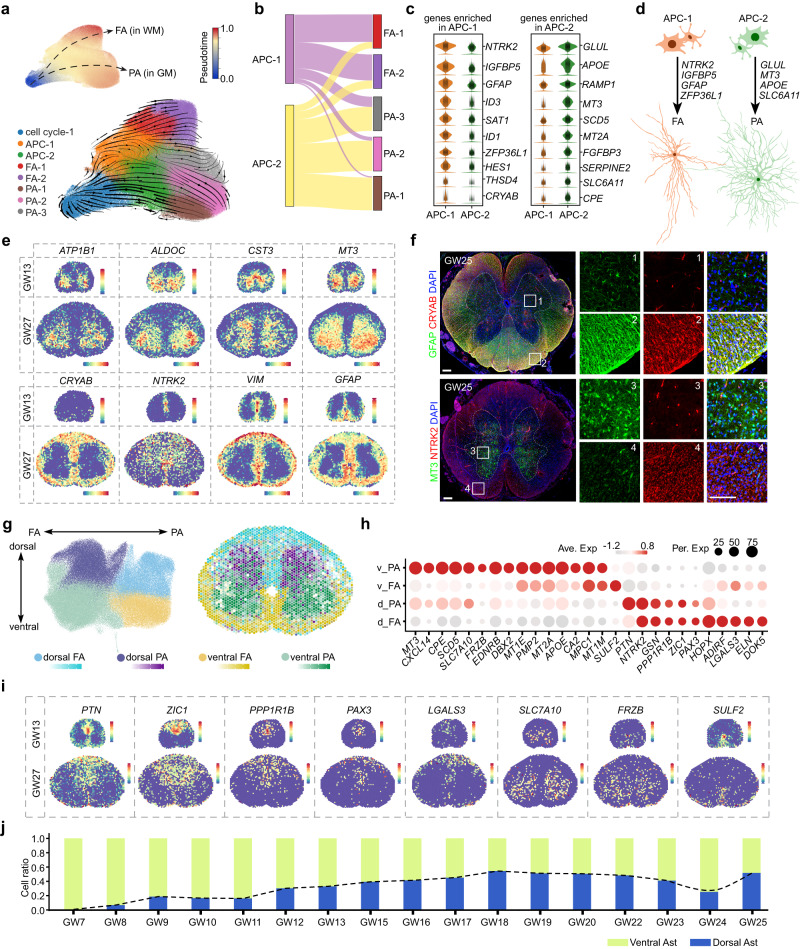
The spatial heterogeneity of astrocytes in the developing human spinal cord. **a** The developmental trajectory of astrocytes in the human spinal cord is analyzed using RNA velocity analysis, incorporating cell cycle-1, APCs, and astrocytes. Subtypes of APC (APC-1 and APC-2) and astrocytes (FA-1, FA-2, PA-1, PA-2, and PA-3) are identified based on characteristic gene expression, and the pseudotime of astrocyte cells is displayed in the upper right corner. **b** Pairwise integration determines the potential for differentiation into distinct astrocyte subtypes, as illustrated by a Sankey plot. **c** The specific gene enrichment in APC-1 and APC-2 cells is shown via violin plots. **d** Schematic depiction of the molecular programs that govern the differentiation of APC-1 into FA and APC-2 into PA. **e** The spatial distribution profiles for FA-specific and PA-specific genes are visualized through the 10× Genomics Visium platform. **f** The immunostaining results demonstrate the selective expressions of CRYAB and GFAP in the white matter, as well as the distinctive expression pattern of MT3 in the gray matter. Scale bars, 200 μm (left), 20 μm (right). **g** The heterogeneity of astrocytes is determined based on their positional allocation and fibrous or protoplasmic properties, and is demonstrated through UMAP embedding and spatially visualized using the 10× Visium datasets of GW27. **h** A dotplot depicting the distinct gene expression profiles of the four fundamental astrocyte groups is displayed. **i** The spatially resolved expression patterns of genes with dorsal and ventral preference are presented in a 10× Visium dataset. **j** A histogram is presented to show the ratios of ventral and dorsal astrocytes during various developmental stages. The dynamic of the cell ratios of dorsal astrocytes is indicated by the dotted line.

Insights into the regulatory mechanisms that govern astrocyte heterogeneity in the human spinal cord remain limited. To address this, we integrated cell cycle-1 cells with molecular cues indicative of astrocytic fate, along with astrocytes and APCs. Employing unsupervised clustering, we identified two distinct groups of APCs, APC-1 and APC-2, as well as five subclusters of astrocytes (Fig. 6a; Supplementary information, Table S9). Then, based on the expression of *SLC1A2* and *GFAP*, marker genes for protoplasmic astrocytes (PA) and fibrous astrocytes (FA), respectively, we annotated the five distinct astrocyte types as FA1–2 and PA1–3 (Fig. 6a; Supplementary information, Fig. S6a). To further investigate how FA and PA diverge during development, we inferred the differentiation trajectory of astrocytes in the human spinal cord at a transcriptomic level, using RNA velocity analysis. We observed that cell cycle-1, as the starting cells, differentiated into APC-1 and APC-2, which then gave rise to FA and PA, respectively (Fig. 6a). This is consistent with the results of pairwise integration of APCs and astrocytes, as illustrated in a Sankey plot (Fig. 6b). These findings suggested that FA and PA originate from distinct groups of progenitors, which led us to investigate the genetic programs that are differentially active in APC-1 and APC-2 (Fig. 6c). Our analysis revealed the specificity of *NTRK2*, *GFAP*, *ZFP36L1*, and *IGFBP5* expression in APC-1, and the distinctive expression of *GLUL*, *MT3*, *APOE*, and *SLC6A11* in APC-2, providing critical insights into the molecular codes that guide different types of astrocyte differentiation (Fig. 6c, d; Supplementary information, Fig. S6a). Furthermore, we identified novel genes that define FA and PA, such as the specific expression of *CRYAB*, *SAT1*, and *ID3* in FA, and *MT3*, *ALDOC*, *CST3*, and *ATP1B1* in PA, which is validated through spatial transcriptome dataset and immunostaining results (Fig. 6e, f; Supplementary information, Fig. S6a–c). Taken together, our findings demonstrated that FA and PA in the human spinal cord arise from separate cell lineages with distinct gene expression profiles.

To explore the spatial characteristics of astrogenesis in the human fetal spinal cord, we conducted unsupervised clustering of astrocytes and identified four distinct groups with unique transcriptomic profiles: dorsal FA, dorsal PA, ventral FA, and ventral PA (Fig. 6g, h). Using the 10× Visium data from GW27, we spatially visualized these transcriptionally distinct astrocyte subtypes (Fig. 6g). Intriguingly, we discovered that some of the genes involved in specifying the spatial fates of neurons, such as *PAX3* and *ZIC1*, were also expressed in astrocytes, suggesting that the spatial specification of neurons and astrocytes may, to some extent, share common transcriptional codes (Figs. 6h, i, 2f; Supplementary information, Fig. S6d). Additionally, we analyzed the ratio of dorsal and ventral astrocytes across different developmental stages and observed that ventral astrocytes were first detectable at early embryonic stages. The ratio of dorsal astrocytes subsequently increased and reached rough equivalence with ventral astrocytes at approximately GW16 (Fig. 6j). These findings provided important insights into the spatial dynamics of astrocyte development in the human fetal spinal cord and suggested potential connections between the development of astrocytes and neurons.

### Unraveling the molecular basis for cell type susceptibility in ALS

**Fig. 7:**
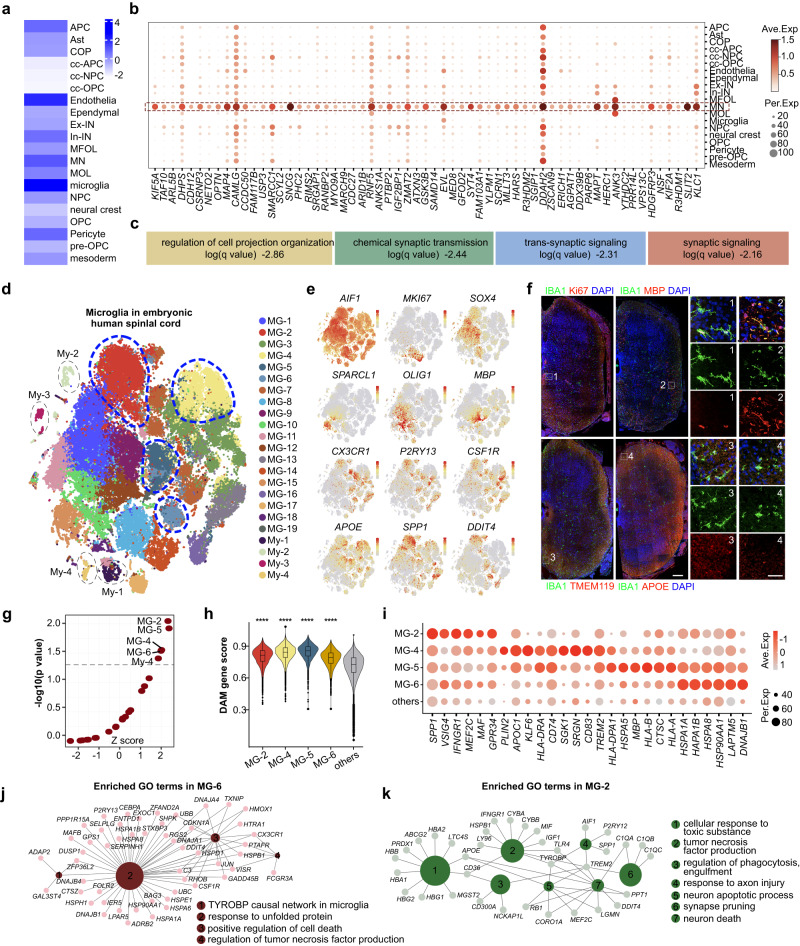
Joint GWAS analysis reveals cell type-specific features of ALS pathogenesis. **a** The susceptibility of different cell types in the human spinal cord to ALS was assessed through the calculation of Z scores using the scDRS method, which involved an integrated analysis of the scRNA-seq dataset of the developing human spinal cord and the ALS GWAS dataset. **b** The differential expression of MN-enriched risk genes associated with ALS, identified through GWAS, in different cell types is depicted in a dotplot. **c** Enriched GO terms attributed to risk genes enriched in MNs are presented. **d** The heterogeneity of microglia in the developing human spinal cord is visualized through *t*-distributed stochastic neighbor embedding (*t-*SNE). Each dot in the plot represents a single cell and is colored according to its cluster identity. **e** The gene expression profiles of heterogeneous microglia subclusters are displayed, with each dot representing an individual cell colored by the expression level (gray for low and red for high). **f** Heterogeneous microglial populations are discernible in the developing human spinal cord at GW16, as ascertained by immunostaining. Scale bars, 200 μm (lower left), 100 μm (right). **g** The relationship between diverse microglial subtypes and ALS risk loci identified through GWAS is established using scDRS analysis, with a significance threshold of *P* < 0.05 indicated by the dotted line. **h** Analysis of DAM gene scores reveals transcriptomic similarity between distinct microglia types and DAMs. Notably, MG-2, MG-4, MG-5, and MG-6 exhibit significantly higher scores compared to other microglia subtypes. **i** Dot plot depicting the characteristic gene expression profiles of microglia subtypes MG-2, MG-4, MG-5, and MG-6. **j**, **k** The GO terms and genetic networks enriched in microglia subtypes MG-2 and MG-6 have been analyzed based on their top 50 DEGs.

ALS is a devastating neurodegenerative disorder characterized by a progressive loss of MNs, resulting in muscle weakness, atrophy, paralysis, and ultimately respiratory failure and death.^29^ Despite significant efforts, the underlying molecular mechanisms of ALS pathogenesis remain incompletely understood. To address this question, we performed an integrated analysis of our scRNA-seq dataset of the developing human spinal cord with a GWAS dataset that included 29,612 ALS patients and 122,656 controls.^30^ We applied the single-cell disease relevance score (scDRS)^31^ to evaluate polygenic disease enrichment of individual cells in scRNA-seq data. Our analysis revealed that microglia, endothelial cells, MNs, and pericytes were the most vulnerable cell types to the ALS risk loci identified by GWAS (Fig. 7a; Supplementary information, Table S10). By intersecting the ALS gene list with DEGs in these four cell types, we identified ALS risk genes specifically enriched in these four vulnerable cell types: MNs (157 genes), microglia (70 genes), endothelial cells (92 genes), and pericytes (29 genes). Given that MN dysfunction and death are the primary hallmarks of ALS, we first focused on the ALS risk genes that are enriched in MNs (Fig. 7b; Supplementary information, Fig. S7a). Among these MN-enriched ALS risk genes, the roles of genes such as *MAPT*, *OPTN*, *NRXN3*, *UNC13A*, and *KIF5A* in ALS have been investigated in previous studies,^32–40^ providing evidence to support our analysis. Our GO enrichment analysis showed that the risk genes that were enriched in MNs were mainly involved in processes such as the regulation of cell projection organization, chemical synaptic transmission, trans-synaptic signaling, and synaptic signaling (Fig. 7b, c; Supplementary information, Fig. S7a).

In addition to MNs, our investigation offered insights into the involvement of non-neuronal cells, particularly microglia, in the pathogenesis of ALS, consistent with the non-cell-autonomous pathology of this disease (Fig. 7a). As the primary resident immune cells of the central nervous system, microglia exhibit immunological properties that can be either neuroprotective or neurotoxic, depending on the circumstances.^41,42^ While growing evidence suggests a role for microglia in ALS, their precise contribution to pathology remains unclear. To better understand the microglial landscape in the developing human spinal cord, we characterized heterogeneous microglial types with unique molecular signatures. These included proliferative microglia expressing *MKI67*, microglia expressing genes indicative of a homeostatic state such as *CX3CR1*, *TMEM119*, and *CSF1R*, and microglia potentially in an active state with genetic characteristics like *APOE*, *SPP1*, and *DDIT4* (Fig. 7d–f; Supplementary information, Fig. S7c). Furthermore, we pinpointed microglia expressing the oligodendrocytic marker *MBP* (Fig. 7e; Supplementary information, Fig. S7c), which has also been documented in the developing white matter of the mouse brain as well as in the mouse spinal cord in other studies.^43–45^ Immunostaining for IBA1 and MBP further provided evidence for demonstrating the presence of MBP^+^ microglia in the developing human spinal cord with preferential distribution in the white matter (Fig. 7f; Supplementary information, Fig. S7c). In addition to diverse microglial types, we identified four myeloid clusters expressing myeloid cell markers such as *LYZ*, *LYVE1*, *S100A4*, and *S100A9* (Supplementary information, Fig. S7b). Our goal was to investigate which microglial subtypes are preferentially involved in ALS pathology. ScDRS analysis revealed that MG-2, MG-4, MG-5, MG-6, and My-4 are significantly associated with GWAS risk loci, suggesting their prioritized involvement in ALS pathology (Fig. 7g).

Previous studies have identified a subset of microglia with unique transcriptional signatures, called DAM, in neurodegenerative diseases such as ALS and Alzheimer’s diseases.^46,47^ DAMs are characterized by the downregulation of homeostatic gene signatures and upregulation of genes involved in phagocytosis, lipid metabolism, and the lysosomal pathway.^46,47^ We thus wondered whether the vulnerable microglial groups (MG-2, MG-4, MG-5, and MG-6) identified in the developing human spinal cord shared some transcriptomic characteristics with DAMs by evaluating the DAM gene score. Our results showed significantly higher scores for MG-2, MG-4, MG-5, and MG-6, suggesting the transcriptional resemblance between DAMs in adult brains and DAM-like microglia residing in embryonic human spinal cord (Fig. 7h). The presence of DAM-like microglia in developing human spinal cord is confirmed by our immunostaining results, where the microglial marker IBA1 co-localizes with DAM-specific markers such as APOE (Fig. 7f), DDIT4, and SPP1 (Supplementary information, Fig. S7c). These results also highlighted the spatial preference of DAM-like microglia in the white matter of the developing human spinal cord (Supplementary information, Fig. S7c). Then, further analysis of our scRNA-seq dataset indicated that the emergence of DAM-like microglia occurs during developmental stages spanning from GW16 to GW25 (Supplementary information, Fig. S7d). The presence of DAM-like microglia in the developing brains of healthy individuals was also reported in previous studies,^43,48–52^ which is in consistence with our findings.

In-depth investigation of the genetic characteristics of the four DAM-like microglial groups through DEG (Fig. 7i; Supplementary information, Table S11) and GO analyses revealed their engagement in activities such as TYROBP causal network in microglia, responding to unfolded proteins, positively regulating cell death, responding to toxic substances, responding to axon injury, participating in neuron apoptotic processes, and activating T cells (Fig. 7j, k; Supplementary information, Fig. S7e). These findings shed light on the role of microglia in contributing to ALS pathogenesis. Taken together, our investigation identified four transcriptionally heterogeneous DAM-like microglia in the embryonic developing human spinal cord, providing valuable insights into the microglial profiles underlying ALS pathogenesis.

Previous investigations have established that the breakdown of the blood-brain barrier and blood-spinal cord barrier is a pivotal factor in ALS and have classified ALS as a neurovascular disease.^53,54^ Our study has also identified endothelial and pericyte cells as the principal cell types associated with genetic risk loci for ALS (Fig. 7a). The risk genes that are specifically expressed in endothelial cells and pericytes were identified by intersecting the annotated risk genes with their DEGs (Supplementary information, Fig. S7a). GO analysis revealed that the endothelial-enriched risk genes are implicated in processes such as angiogenesis, hemostasis, and cell junction organization, whereas the pericyte-enriched risk genes are associated with vasculature development, among other processes (Supplementary information, Fig. S7f). Thus, our findings regarding the involvement of endothelial cells and pericytes in ALS and the associated risk gene expression preferences in these cell types may provide insight into the neurovascular causes of ALS. Taken together, our results shed light on the possible cellular mechanisms of ALS and risk gene expression preferences in specific cell types, which is insightful to our understanding of disease pathogenesis and could inform the development of precise therapeutic treatments.

## Discussion

The spinal cord plays a crucial role in sensory perception and motor response by construction of functionally complex neuronal circuits, which are regulated in a strictly spatial and temporal manner by both extrinsic and intrinsic systems. Decades of research in model organisms have identified the pivotal roles of TFs in defining and instructing the generation of diverse neuronal populations within the spinal cord.^7,8,55–58^ Combinatorial TF transcriptional control of cell fate is now a mature perspective for understanding spinal cord development. However, the spatiotemporal TF codes for neuronal specification in the human spinal cord remain unclear. To address this knowledge gap, we have generated a comprehensive transcriptional repertoire of the developing human spinal cord. Our datasets span embryonic stages ranging from the early first to third trimesters, providing invaluable insights into the temporal transcriptional dynamics over almost the entire embryonic stages. We have also adopted two types of spatial transcriptome datasets, which offer spatially-resolved gene expression information that is essential in delineating the specific gene expression in specific cells at specific locations. Furthermore, we have developed an image-based single-cell spatial transcriptome method, TF-seqFISH, to detect the spatially restricted expression of 1085 TFs in the developing human spinal cord. Although transcriptomic datasets of developing human spinal cords already exist,^14,59–61^ our TF-seqFISH dataset provides an invaluable source for elucidating the characteristic spatial TF expression in developing human spinal cords. Based on our TF-seqFISH dataset, we elucidated the characteristic spatial organization of VZ-resident NPCs along a dorsoventral axis, as well as the sandwich-like spatial assignments of Ex-INs and In-INs in the dorsal horn of the developing human spinal cord at early embryonic stages. The TF-seqFISH also enabled us to unravel the mediolateral assignments of TFs that are crucial for MN differentiation and specification. The TF spatial regulation of MN diversification is also demonstrated by the differential TF expression along the rostrocaudal axis, which is the molecular basis for forming MN columns and pools. In addition, we identified five molecularly demarcated regions in the GW8 spinal cord slice based on our TF-seqFISH dataset, providing a molecular explanation for its coincident anatomical demarcation. All of these results demonstrate that TF-seqFISH is a reliable and powerful method for studying spinal cord development. Given the importance of TFs in shaping the spinal cord and brain development, our TF-seqFISH method is undoubtedly a valuable tool for researchers.

In this study, we elucidated the early initiation of neurogenic and gliogenic events in the developing human spinal cord, which may occur even earlier than GW6, considerably earlier than those observed in the human brain. Despite newborn babies appearing helpless and having limited motor abilities compared to other animals, they already possess the capacity to move their head, trunk, and extremities for several months before birth.^62^ By GW7, the first movements of the fetus can be detected by ultrasound examinations.^63^ To ensure accurate perception of extrinsic and intrinsic signals and basic motor responses during development, the sensory and motor connections need to develop early. Myotome differentiation, which forms the muscles, begins at week 5,^64^ and sensory and motor connections develop during this time to establish the first elementary reflex circuitries.^65^ Thus, the early neurogenic and gliogenic events in the developing human spinal cord are likely driven by fetal demands for early activities.

Previous research in mice and chicks has demonstrated that NPCs in the developing spinal cord can be divided into distinct subtypes along a DV axis, with each subtype giving rise to functionally distinct neuronal types.^1,5–7,66^ Building on these findings, we used combinatorial TF expression patterns observed in mice as a reference to identify and characterize molecularly distinct NPC subtypes in the developing human spinal cord. Using our TF-seqFISH dataset, we observed that the spatial position of NPC subtypes along the DV axis in the human spinal cord is similar to that reported in mice. However, we also found gene expression differences along the DV axis, such as the widespread expression of *PTF1A* in dp3–6 cells in the developing human spinal cord, in contrast to its restriction in dp4 in mouse spinal cord. Moreover, we also investigated the spatial underpinnings of astrocyte development in the human spinal cord. Our results reveal a strong synergy between astrocyte and neuronal development, as both of which advancing in the ventral part. Furthermore, the transcriptional codes that are responsible for the spatially resolved specification of neurons and astrocytes exhibit parallel patterns, to some extent. These findings are important for understanding the neurogenic and gliogenic events in the developing human spinal cord. Limited endogenous neurogenesis and axon regeneration have been observed following repairing spinal cord injuries (SCI), which may be due to a failure to recapitulate the spatiotemporal developmental processes. Our study provides insight into the spatiotemporal codes underlying neuronal and glial augmentation in the human spinal cord, which could inform stem cell therapy approaches to repair SCI.

Sensory perception and discrimination of various stimuli are critical for animal survival. The ability to distinguish between noxious and innocuous stimuli is primarily dependent on the dorsal horn circuitry in the spinal cord. In mammals, the dorsal horn neurons are organized in a characteristic dorsoventral laminar pattern that facilitates the processing of different sensory modalities, including pain, temperature, touch, and itch. However, there is a dearth of systematic studies investigating the development of this circuitry in humans. Here, we presented an integrated analysis of scRNA-seq and spatial transcriptome data to unravel the cellular and molecular rules that underlie dorsal horn development in the human spinal cord. Our findings demonstrated the existence of early and late waves of neurogenesis before and after GW8, respectively. Furthermore, we showed that the early-born neurons (dI1–4) and late-born neurons (dI5–6) are destined for the deep and superficial lamina of the dorsal horn, respectively, while their progenitor cells (dp1–dp6) are ordered sequentially along a dorsoventral axis. Thus, this spatial pattern requires the ventrolateral and dorsolateral migration of early- and late-born neurons, respectively, to achieve their distinct laminar positions, thus establishing the DV laminar pattern of the dorsal horn.

Remarkably, we also observed a distinctive sandwich-like spatial arrangement of Ex-INs and In-INs within the dorsal horn at GW8, which is consistent across the entire spinal cord. However, by GW9, this sandwich-like structure has transitioned into a laminar organization in the dorsal horn, as revealed by our 10× Visium dataset, indicating the disappearance of the sandwich-like structure at this stage. This observation leads us to propose that the sandwich-like spatial arrangement of Ex-INs and In-INs reflects an intermediate state formed by temporal neurogenetic events and spatially characterized neuron migration within the dorsal horn. Specifically, In-INs located near the VZ likely represent nascent late-born neurons, interspersed with newly generated Ex-INs. Conversely, laterally positioned In-INs may correspond to early-born neurons that have engaged in lateral migration. Lastly, Ex-INs situated between them could be early-born Ex-INs that migrated ventrally to occupy the deeper lamina of the dorsal horn. Interestingly, a similar transient sandwich-like structure was also evident in the spinal dorsal horn of mice at E11–E12. In the studies by Muller and Gross et al., they identified a bifurcate pattern of Lbx1^+^ cell distribution. Furthermore, their findings demonstrated the ventral migration of Isl1^+^ Ex-INs, which are sandwiched between Lbx1^+^ cells. They also illustrated that at E15.5, the Pax2^+^ In-INs and Lmx1b^+^ Ex-INs cells are intermingled in the dorsal horn with canonical lamination organization.^67,68^ These results indicate that the sandwich-like arrangement of neuronal populations might be a shared characteristic during spinal dorsal horn development across species. This arrangement seems to emerge temporarily during the establishment of lamina organization, which is significant for advancing our comprehension of the mechanisms governing the development of the dorsal horn.

Apart from the potential conserved traits governing the dorsal horn development, the alignment between human and mouse spinal cord development is also evident in the molecular and spatial organization of the VZ-resident NPCs in the developing spinal cord, particularly concerning TF expression. This similarity extends further to the shared molecular principles underlying MN diversification. Although some genetic differences between human and mouse spinal cord cells have been reported,^14,59,69^ our results suggested that the general strategies underlying spinal cord development might be conserved between these two species.

ALS is a devastating neurodegenerative disorder characterized by progressive loss of MNs, resulting in muscle weakness, paralysis, and ultimately death of the patient.^70^ Unveiling the pathogenesis of ALS to identify effective treatments has been a challenging task for researchers. In our study, we employed scRNA-seq datasets of the developing human spinal cord to map GWAS risk loci of ALS and identified the cell types transcriptionally vulnerable to ALS and the underlying molecular regulation. Specifically, we identified the early transcriptomic specification of MN diversity and its relevance to ALS. However, it is important to acknowledge that ALS is generally characterized by adult-onset symptoms, and the transcriptional profiles of embryonic MNs may differ from those of their adult counterparts. Further experimental validation is needed to definitively establish the precise correspondence between the embryonic MN subtypes and their adult counterparts. In addition to MNs, we identified the involvement of microglia, endothelial cells, and pericytes in ALS by assessing risk loci enrichment in distinct cell types, in line with the non-cell-autonomous pathology of ALS. Notably, we have identified a subset of DAM-like microglia exhibiting molecular characteristics similar to the DAMs that are active in neurodegenerative disorders, and are detected as the ones preferentially involved in ALS. The DAM-like microglia have also detected in the developing brains of healthy individuals in previous studies,^43,49–51,71^ updating our understanding of microglia heterogeneity in the embryonic central nervous system.

In our study, the DAM-like microglia identified in the developing human spinal cord exhibit remarkable similarities to the specialized microglial populations observed in several studies.^43,48,49^ First, both types of DAM-like microglia display an up-regulation of disease-associated genes, concomitant with a downregulation of the homeostatic gene cassette. Second, both types of DAM-like microglia predominantly emerge within developing white matter regions, such as the corpus callosum and cerebellar white matter in earlier studies^43^ or the white matter of the spinal cord in our analysis. Third, the occurrence time of the DAM-like microglia identified in the developing human spinal cord (GW16–25) or in previous studies both aligns with the onset of myelination. Fourth, consistent with the spatial distribution of DAM-like microglia, we also detected abundant MBP^+^ microglia in the white matter of the spinal cord. Thus, drawing from the transcriptional and spatiotemporal similarities between the DAM-like microglia identified in our study and those reported in other research, along with the functional analysis of early postnatal DAM-like microglia in previous studies, we hypothesize that the DAM-like microglia present in the developing spinal cord might play pivotal roles in regulating the myelination process during human spinal cord development. It is plausible to speculate that DAM-like microglia could potentially influence the myelination of MN processes, thereby contributing to the establishment of proper neural circuitry. However, further investigations are necessary to ascertain the precise functions of DAM-like microglia in the developing spinal cord. Given the challenges of directly focusing on MN regeneration, our study offers alternative choices to regulate microglial activities or neurovascular systems, which may hold promise for treating ALS in the future.

## Materials and methods

### Human subjects

The de-identified human tissue collection and research protocols were approved by the Reproductive Study Ethics Committee of Beijing Anzhen Hospital (2014012X) and the institutional review board (ethics committee) of the Institute of Biophysics (H-W-20170110). The fetal tissue samples were collected after the donor patients signed an informed consent document. All experiments were carried out in accordance with protocol guidelines.

Human fetal spinal cord samples obtained from 10 donors, ranging from GW7 to GW25 were carefully collected and processed to generate single-cell cDNA libraries. The samples were dissected into cervical, thoracic, and lumbar segments before being dissociated into a single-cell suspension using a papain-based dissociation protocol in ice-cold artificial cerebrospinal fluid. As a result, our scRNA-seq dataset comprises a total of 30 samples. The resulting single cells were then suspended in 0.04% BSA/PBS at an appropriate concentration to generate cDNA libraries with Single Cell 3’ Reagent Kit (10× Genomics, 1000268). During library preparation, enzymatic fragmentation and size selection were performed to optimize cDNA size, and P5, P7, an index sample, and R2 (read 2 primer sequence) were added to each selected cDNA during end repair and adaptor ligation. The cDNA libraries were then processed on the Illumina platform for sequencing with 150 bp pair-end reads. Overall, this careful and precise methodology allowed us to generate high-quality single-cell cDNA libraries, which are critical for the accurate and reliable analysis of the cellular and molecular mechanisms underlying spinal cord development.

### 10× Visium spatial transcriptomics

Fresh human developmental spinal cord tissues were snap-frozen in Optimal Cutting Temperature (O.C.T.) compound (SAKURA) after embedding and stored in dry ice-ethanol mixture. Subsequently, tissue blocks were sectioned into 10 μm sections using a cryostat (Lecai CM3050 S) and placed on Tissue Optimization Slides and Gene Expression Slides (10× Genomics). Our study utilized the cervical segmental sections from developing human spinal cord at different gestational stages, including 18, 15, 9, and 3 sections from GW8, GW9, GW13, and GW27, respectively, for conducting 10× Visium experiments. These tissue sections were obtained from a total of 5 donors, with the GW13 sections originating from two of these donors. Thus, our 10× Visium analysis encompassed a total of 45 tissue sections derived from 5 donors. The reproducibility data for the tissue sections is presented in Supplementary information, Fig. S4a.

The optimal permeabilization time was determined by following the manufacturer’s instructions (10× Genomics, Visium Spatial Tissue Optimization, CG000238 Rev D), with different times chosen for different tissues. H&E-stained sections were prepared using the manufacturer’s protocol (10× Genomics, Methanol Fixation, H&E Staining & Imaging for Visium Spatial Protocols, CG000160 Rev B) and imaged using an Olympus FV3000 imaging system with Olympus DP80 CCD camera and an Olympus 10×/0.40 objective.

Subsequently, the tissue sections were subjected to gene expression analysis using the manufacturer’s protocol (10× Genomics, Visium Spatial Gene Expression Reagent Kits, CG000239 Rev D). Briefly, H&E-stained sections were permeabilized, followed by reverse transcription, second-strand synthesis, and denaturation. qPCR analysis was performed using the KAPA SYBR FAST kit (KAPA Biosystems) and the QuantStudio 6 Flex system (ThermoFisher Scientific). The cDNA amplification cycle number was determined based on ~25% of the peak fluorescence value. The final libraries were sequenced on the Illumina HiSeq Xten system using 150 bp pair-end reads.

### TF-seqFISH

#### Probe library design

To enable high-throughput in situ hybridization for the investigation of TFs in human tissue samples, we developed a probe library consisting of 1085 mRNA-specific probes, each designed to include a 28-nt mRNA complementary sequence, four 18-nt readout sequences, and two primer sequences to allow for PCR amplification. To ensure optimal imaging quality, we aimed for each barcoded mRNA to bind with 17–32 probes, with binding regions preferentially selected from exons. To avoid off-target effects, we employed a local BLAST program to screen the selected sequences, removing any with 17-nt or more of homology to non-target sequences. The randomly generated readout sequences were designed to have a GC fraction ranging from 40%–60% and were subjected to a local BLAST against the human genome to remove any non-specific sequences. Finally, any sequences with a 10-nt or greater match were excluded from the probe library. Our library was designed based on the hg38 release 97 version of the human genome and annotation, which we obtained from the Ensembl FTP server. Overall, our probe library design aimed to optimize specificity and sensitivity, ensuring reliable detection of transcription factor gene expression in human tissue samples.

#### Probe construction

We developed gene-targeted probes utilizing a complex oligonucleotide pool. To accomplish this, we conducted a limited-cycle PCR amplification of the custom-designed oligopool to generate dsDNA templates that were subsequently used as templates for in vitro transcription facilitated by T7 RNA polymerase. Reverse transcription was performed to generate single-stranded DNA (ssDNA) probes. To further refine the probes, the mixture of reverse transcription products was treated with a uracil-specific excision reagent (USER) enzyme to remove the 5’ forward primer.^72^ We then utilized alkaline hydrolysis and ethanol precipitation to purify the resulting ssDNA probes from the reaction mix. Readout probes were ordered from Integrated DNA Technologies and were 5′ modified with either Alexa Fluor 647 or Alexa Fluor 488.

#### Imaging sample preparation

The human spinal cord tissues were meticulously prepared for RNA imaging. Firstly, they were fixed in 4% paraformaldehyde and cryoprotected by sinking them in 30% sucrose in 1× PBS. Subsequently, the tissues were embedded in O.C.T. compound and sectioned into 10 μm slices. To prepare the tissue slices for imaging, they were permeabilized using 70% ethanol at 4 °C, followed by treatment with 10% SDS at room temperature. The slices were then hybridized with a targeting probe in a 37 °C oven overnight. Post-hybridization, the slices were washed in 20% formamide at 37 °C and treated with 0.1 mg/mL label-X solution, as previously described.^73^ To improve the imaging quality, the slices were embedded in a 4% hydrogel composed of a 19:1 acrylamide/bis-acrylamide solution and treated with 10 U/mL protease K buffer for digestion.^74^ Finally, the hydrogel slices were stored in 0.4 U/μL RNase inhibitor at 4 °C before imaging.

#### Images acquisition

To visualize gene expression in situ, tissue sections were embedded in hydrogel and attached to a flow-cell chamber of appropriate size. The chamber was connected to an automated fluidics system and a spinning disk imaging system (Dragonfly 500, Andor Technology Ltd). Each hybridization-imaging cycle involved pumping 300 μL of hybridization buffer, containing 50 nM Alexa Fluor 647, 50 nM Alexa Fluor 488, 8% ethylene carbonate, 10% dextran sulfate, and 0.4  U/μL RNase inhibitor in 2× SSC, into the flow-cell chamber. This was followed by addition of 10 μg/μL DAPI solution, 20% formamide washing solution, and anti-bleaching solution (8% (w/v) glucose, 1 mg/mL glucose oxidase, 10 μg/mL catalase, and 0.4 U/μL RNase inhibitor). Images of the 488 and 637 channels for selected fields of view were captured for 22 rounds of hybridization-imaging cycles, while images of the 405 channel were taken for cell segmentation and horizontal-shift correction.

### Immunofluorescence

Human fetal spinal cords were processed for immunofluorescence analysis using standard procedures. Briefly, the spinal cords were fixed in 4% paraformaldehyde in PBS at 4 °C and dehydrated in 30% sucrose in PBS. The fixed and dehydrated tissues were embedded in O.C.T. compound and frozen at –80 °C. Cryosections were cut using a Leica CM3050S and subjected to antigen retrieval and pretreatment with 0.3% Triton X-100 in PBS. The sections were then incubated in a blocking solution containing 10% normal donkey serum, 0.1% Triton X-100, and 0.2% gelatin in PBS, followed by overnight incubation with primary antibodies at 4 °C. The following primary antibodies were used: MNX1 (Abcam, ab221884), VGLUT1 (Millipore, AB5905), GABA (Sigma, A0310), GFAP (Abcam, ab10062), PDGFRA (Santa Cruz, sc-431), MBP (Abcam, ab218011), OLIG2 (Millipore, AB9610), ATOH1 (Proteintech, 21215-1-AP), PAX7 (DSHB), PAX6 (BioLegend, 901301), SOX2 (Santa Cruz, sc-17320), OLIG3 (Abnova, H00167826-B01), ETV1 (Invitrogen, PA5-77975), NKX2-2 (DSHB, 74.5A5), FOXA2 (R&D, AF2400), NKX6-1 (R&D, AF5857), MASH1 (BD, 556604), PAX3 (DSHB, Pax3), CRABP1 (NOVUS, NB300-539), DRGX (NOVUS, NBP1-94059), ISL1 (R&D, AF1837), FOXP1 (Abcam, ab16645), LHX3 (DSHB, 67.4E12), LHX1 (Abcam, ab14554), CRYAB (NOVUS, NB100-2519), NTRK2 (R&D, AF1494), MT3 (Sigma, HPA004011), IBA1 (Abcam, ab5076), TMEM119 (Abcam, ab185333), and APOE (Abcam, ab52607). After incubation with primary antibodies, the sections were washed three times for 10 min with 0.1% PBST and then incubated with secondary antibodies diluted in a blocking solution. The following secondary antibodies were used: donkey anti-mouse 488 (Invitrogen, A21202), donkey anti-rabbit 594 (Invitrogen, A21207), donkey anti-rabbit 488 (Invitrogen, A21206), donkey anti-goat 488 (Invitrogen, A-11055), and donkey anti-chicken 488 (Invitrogen, A78948). The sections were counterstained with DAPI and mounted with mounting medium (Sigma). Immunofluorescence images were acquired using an Olympus laser confocal microscope and analyzed with FV10-ASW viewer (Olympus), ImageJ (NIH), and Photoshop (Adobe).

### Alignment and preparation of scRNA-seq and 10× Visium data

scRNA-seq and 10× Visium data were processed using widely used computational tools. Specifically, Cell Ranger (versions 2.0.1 and 3.0.2) was used for sequence alignment, barcode filtering, and unique molecular identifier (UMI) counting for scRNA-seq data, while Space Ranger (version 1.3.0) was utilized to process, align, and summarize UMI counts for each spot on the Visium transcriptomics chips. The human reference genome (hg19) was used for both types of data. Default parameters were employed during processing and analysis.

### scRNA-seq data analysis

We aimed to construct a comprehensive transcriptional landscape of the developing human spinal cord by combining our own scRNA-seq datasets with that of Zhang et al.^14^ To ensure data quality, we first removed potential doublets using the Scrublet package.^75^ Next, we filtered the UMI matrix, excluding cells that did not meet specific criteria, including a detected number of genes between 600 and 10,000, UMI counts less than 40,000, mitochondrial counts less than 0.2, and hemoglobin counts less than 0.4. After this filtering, we were left with 912,514 cells and 21,660 genes, with 217,636 and 694,878 cells originating from our own datasets and Zhang et al.,^14^ respectively. We then conducted normalization, highly variable gene finding, and principal component analysis (PCA) reduction using the Scanpy package. To correct for batch effects, we utilized the BBKNN algorithm.^76^ Unsupervised clustering was performed using the Louvain algorithm in the Scanpy package, with UMAP for visualization.

### Developmental trajectory inference in the developing human spinal cord

To deduce the developmental trajectory of neurogenic and gliogenic events in the developing human spinal cord, we employed the scVelo package^77^ to compute the raw spliced and unspliced mRNA counts from BAM files for each sample. Subsequently, the velocity vector was computed based on the UMAP layout. In this study, the cell density is evaluated using the embedding_density function in the Scanpy package with its default parameters. The specific steps are outlined below: Firstly, we annotated the cell types associated with the neurogenic, astrocytic, and oligodendrocytic lineages based on the outcomes of unsupervised clustering and classical gene expression analysis. These annotations were further visualized using UMAP.

Subsequently, we employed the ‘scvelo’ Python package to perform RNA velocity analysis. Following the recommendations from the tutorial, we curated gene selection and filtered out genes with shared counts of fewer than 20 between spliced and unspliced matrices. We normalized the data using the scv.pp.normalize_per_cell function and identified the top 2000 most variably expressed genes for further investigation. Next, we applied the scv.pp.log1p function for logarithmic transformation. Utilizing the selected genes, we calculated the first and second moments through the scv.pp.moments function. We inferred cell velocities by modeling transcriptional dynamics using a stochastic model, facilitated by the scv.tl.velocity function.

To project velocities onto a UMAP embedding, we estimated transition probabilities for cell-to-cell transitions with the scv.tl.velocity_graph function. Finally, we visualized cell state transitions by projecting velocities onto a precomputed UMAP using the scv.pl.velocity_embedding_stream function.

### Exploring cell type ratio dynamics during neurogenic and gliogenic events in human cortex development

To elucidate the critical temporal landmarks of neurogenic and gliogenic events during human fetal brain development, we performed a comprehensive analysis of distinct cell type ratios. To obtain a comprehensive and representative overview, we integrated multiple scRNA-seq datasets of the cortex, including those from Bhaduri et al. (2021)^15^ and Nowakowski et al. (2017),^17^ and Polioudakis et al. (2019).^18^ To ensure consistency across studies, we converted the Carnegie Stage and postconceptional weeks to GW using the methodologies outlined in Haniffa et al. (2021)^78^ and O’Rahilly and Muller (2010).^79^ Normalized ratios of cells were calculated for each week, and the locfit function from the locfit package was employed to model the trend of cell type development over time.

### Neuronal lineage determination

To delineate the distinct neuronal lineages responsible for generating different types of neurons, we analyzed scRNA-seq datasets containing NPCs and neurons of three different identities: Ex-Ins, In-INs, and MNs. To reduce computational load, we initially focused on analyzing scRNA-seq datasets derived from our own data. These datasets were subjected to a standard processing pipeline, starting with data normalization using the ‘SCTransform’ function of the Seurat package. Subsequently, PCA reduction was performed, followed by batch effect removal using RPCA integration procedures. Unsupervised clustering was then carried out using the ‘FindClusters’ function of Seurat, and the results were visualized using a UMAP embedding. Following these steps, we mapped the scRNA-seq data of NPCs and neurons from Zhang et al.‘s study^14^ to our own datasets, yielding a combined scRNA-seq dataset that was used to analyze neuronal lineage.

Based on the results of unsupervised clustering and classic marker gene expression, we have successfully classified the NPC cells into distinct groups, namely dp1, dp2, dp3–6, p0/1, p2/3, and pMN, respectively. Additionally, we have also categorized the neurons into dI1, dI2, dI3, dI4, dI5, dI6, V0, V1, V2, MN, and V3, according to their gene expression profiles.

Then, we probed the lineage relationships between these distinct NPCs and neuronal groups by referring to the results of RNA velocity analysis. The RNA velocity analysis was conducted using the ‘scvelo’ Python package. In this analysis, the arrow directions, indicative of differentiation trends, are determined through evaluating the spliced and unspliced RNA states within each cell. The potential root and endpoint cells are estimated using the ‘scv.tl.terminal_states’ function, and pseudotime scores are determined using the ‘scv.tl.velocity_ pseudotime’ function. Hence, the low pseudotime scores assigned to NPCs are determined through an unsupervised approach using the results obtained from the RNA velocity analysis. Based on the results of RNA velocity analysis, our study has revealed the differentiation of dp1 towards dI1, dp2 towards dI2, dp3–6 towards dI3, dI4, dI5, and dI6, p0/1 towards V0 and V1, p2/3 towards V2 and V3, as well as pMN towards MN. To identify DEGs among these distinct cell types, we have employed the FindAllMarkers function in the Seurat package.

### MN development analysis

To investigate MN development in the developing human spinal cord, we subjected scRNA-seq datasets of pMNs and MNs from various samples to a routine pipeline detailed in “Neuronal lineage determination”. Following this processing, pMN and MN cells were visualized in a UMAP embedding and classified into categories including pMN, newly born MN-1, newly born MN-2, LMCl, LMCm, PGC, MMC, and HMC. The velocity vector of MNs was then calculated using scVelo’s stochastic model with default parameters on this UMAP, and trajectory pseudotime was calculated via the velocity_ pseudotime function with default parameters.

To investigate the molecular cues of motor columns that might seed in newly born MNs, we performed CCA integration of newly born MNs and MNs with distinct column identities using RunCCA in the Seurat package. Then, we conducted k-nearest neighbors analysis in CCA space to predict the potential fates of newly born MNs by voting for their nearest MN neighbors using the KNN function in R with parameters k = 5 and l = 4.

### Gene expression change along MN differentiation

To investigate the molecular changes occurring during MN differentiation from pMN to newly born MNs, we employed GAM regression to identify genes with significant expression changes along pseudotime (gene list 1). Subsequently, we determined the DEGs among pMN, newly born MN-1, and newly born MN-2 (gene list 2). We selected genes that were present in the intersection of both lists 1 and 2 for further analysis. Gene expression was then modeled as a smooth function with pseudotime by fitting a vector generalized additive model using the VGAM package.^80,81^ The heatmap visualized the dynamic expression profile of genes along pseudotime.

### Astrocyte development and lineage trajectory inference

To unravel the intricacies of astrocyte development in the developing human spinal cord, we employed a standard pipeline in Scanpy to analyze a scRNA-seq dataset comprising cells of cell cycle-1 (with transcriptionally astrocytic cues), APC, and astrocytes. Through unsupervised clustering and classical marker analysis, we further distinguished APC cells into APC-1 and APC-2, and astrocytes into FA-1, FA-2, PA-1, PA-2, and PA-3. Subsequently, we identified the DEGs among the various cell groups using the FindAllMarkers function in the Seurat package. The RNA velocity analysis was conducted using the scVelo package, employing a stochastic model with default parameters, to infer the astrocytic differentiation trajectory. Furthermore, we integrated APCs and astrocytes using DEGs through CCA integration and applied the KNN algorithm (k = 10, replication_times=5000)^82^ as an alternative method to determine the differentiation potential of APC-1 and APC-2 cells. Our parallel analyses revealed the differentiation preference of APC-1 and APC-2 towards FA and PA, respectively.

### Determining the spatial identities of astrocytes

To unveil the spatial information encoded in astrocytes, we scrutinized scRNA-seq datasets of FAs and PAs. First, we corrected for batch effects across samples by implementing the BBKNN algorithm in Scanpy.^76^ Next, we performed unsupervised clustering with the Leiden algorithm and visualized the results using UMAP. Guided by the clustering and expression of classic markers, we discriminated four distinct clusters comprising dorsal and ventral PAs, as well as dorsal and ventral FAs. To obtain a spatial representation of these astrocyte subtypes, we employed the Tangram algorithm^83^ to project the scRNA-seq data onto our 10× Visium dataset using the cluster mode. Tangram leveraged the top 300 DEGs of each cell type to project the cell types from the scRNA-seq datasets onto the 10× Visium datasets. The resulting normalized probability of the presence of each astrocyte subtype in the 10× Visium datasets was displayed.

### Identification of DEGs among clusters

We utilized the Seurat package to conduct differential gene expression analysis among the clusters, employing the FindAllMarkers function. Genes displaying an adjusted *P* value below 0.05 were deemed significant and thus identified as DEGs.

### 10× Visium data analysis

We employed the SCTransform method in the Seurat package to normalize the data and identify variable genes. The cells were then integrated using the Seurat RPCA integration algorithm (with 3000 features and default parameters). Unsupervised clustering was carried out using the FindClusters function in Seurat. To enable visualization, we used the UMAP algorithm to reduce the dimensionality of the data (first 30 PCs as input, min.dist = 0.3, spread = 1, n.neighbors = 30). The clusters were annotated by considering the combinatorial expression of their DEGs and spatial location.

### Integration of scRNA-seq data with 10× Visium data

To elucidate the neuronal identities of both early- and late-born neurons in the 10× Visium dataset, we employed the Tangram algorithm to map the scRNA-seq datasets onto the Visium data. In essence, we selected the top 100 DEGs for each cell type as training genes for Tangram. This allowed us to project scRNA-seq cell types onto the Visium data, where we utilized normalized probabilities for data visualization.

### Analysis of TF-seqFISH data

Image registration was performed by aligning each round to the first round using DAPI images. To eliminate uneven illumination effects, we applied the tophat algorithm, followed by the ROF algorithm to remove background noise. The Richardson-Lucy deconvolution algorithm was then applied to images to enhance the signal. For cell segmentation, a deep neural network based on ResUNet was trained, which took DAPI and Nissl images, as well as manual labels as input. The resulting output was post-processed with the watershed algorithm to obtain the final cell boundary. We located potential mRNA transcript signals by identifying local maxima in the processed image above a predetermined pixel threshold. To further resolve spot locations, we calculated the radial center of the 2D intensity distribution. Once all spots were obtained, we found the nearest neighbor spots within a 2.5-pixel radius for each spot in the other three barcode channels. If the spot combinations matched a single barcode, these spots were added to the barcode set. For spots that matched multiple barcodes, we kept the spot sets with the minimum distances and matched the spots to the closest target barcode. Ambiguous spots were dropped if multiple barcodes were still possible. We repeated this procedure using each of the four barcode channels as a seed and only barcodes that were called in at least three of four rounds were validated as genes. For downstream analysis, we used the SCTransform function in the Seurat package to normalize data and select variable genes. Dimension reduction was performed using the RunPCA function, and clustering was done using the FindClusters function in Seurat. We used the UMAP algorithm with default parameters to visualize cell clusters.

### Integration analysis of the scRNA-seq and TF-seqFISH datasets

To investigate the spatial organization of distinct NPC groups (dp1, dp2, dp3–6, p0–1, pMN, and p3) along the dorsoventral axis, we performed an integrated analysis of the TF-seqFISH data and the scRNA-seq data obtained from GW8 to ensure temporal alignment. To establish the correspondence between the two datasets, we identified highly variable genes in the TF-FISH data that were also expressed in the scRNA-seq data and used them as integration features. We then used the FindTransferAnchors function in Seurat to identify a set of anchors between the two datasets. Finally, we obtained the predicted identity of each TF-FISH cell based on probabilities calculated using the TransferData function, with prediction.assay set to TRUE.

### Spatial gene imputation

To estimate the expression levels of genes that were not directly profiled by the TF-seqFISH technique, we utilized the Seurat transfer continuous data pipeline, which leverages scRNA-seq data as a reference. We first identified anchors between the scRNA-seq and FISH data using the FindTransferAnchors function, and then used the TransferData function to impute the gene expression levels based on the normalized scRNA-seq expression data.

### Joint analysis with GWAS

To investigate the cellular targets of ALS, we applied a cell-specific vulnerability scoring approach using the scDRS package (version 1.0.3). The scDRS algorithm, as introduced by Zhang et al.^31^ represents an innovative method that connects scRNA-seq data with polygenic disease risk at single-cell resolution. The process involves the following steps: (1) Construction of Disease Gene Set: Using summary statistics from a disease GWAS, scDRS identifies a set of putative disease genes. (2) Calculation of Score: For each cell in the scRNA-seq dataset, scDRS computes a raw disease score based on the expression of the genes in the disease gene set. scDRS generates B Monte Carlo samples of raw control scores for each cell. (default B = 1000). (3) Normalization and *P* value Calculation: Normalization is performed at both gene set and cell levels. scDRS then calculates an association *P* value for each cell by comparing its normalized disease score with the empirical distribution of normalized control scores. *P* values can also be used for data visualization and statistical inference.

In the context of our study, we leveraged the comprehensive summary statistics from a recent large-scale GWAS of ALS patients.^30^ The SNP2GENE function was utilized to upload the GWAS summary statistics file with default parameters (sample size = 152,268, lead SNP *P* value threshold ≤ 5^e–8^, *P* value threshold ≤ 0.05, ${r}^{2}$ of independent significant SNPs ≤ 0.6, 2nd ${r}^{2}$ of lead SNPs ≤ 0.1, and distance between LD blocks to merge into a locus ≤ 250 kb). The FUMA pipeline was employed to annotate single nucleotide polymorphisms and map variants to genes within 10 kb of lead SNPs. MAGMA, integrated into FUMA, generated Z-scores reflecting gene association with ALS. The top 1000 genes with the highest Z-scores, indicative of strong ALS association, were selected to form the ALS gene list.

Utilizing this ALS gene list and its associated weights, along with scRNA-seq data, the scDRS algorithm was employed to calculate cell type-specific vulnerability scores. Specifically, four clusters (microglia, endothelial cells, MNs, and pericytes) were identified as potential targets of ALS based on scDRS results. By intersecting the ALS gene list with DEGs in these four cell types, we identified ALS risk genes specifically enriched in each category: MNs (157 genes), microglia (70 genes), endothelial cells (92 genes), and pericytes (29 genes). This approach provides a nuanced understanding of cell type-specific ALS genetics, thereby shedding light on underlying mechanisms of cellular vulnerability.

### Microglia heterogeneity identification

To characterize microglial heterogeneity, we isolated all microglial cells from the entire scRNA-seq dataset for comprehensive analysis. Following normalization, variable feature selection, and PCA reduction, as described in the “Neuronal lineage determination” section, *t-*SNE was employed to enhance the visualization of the data. The FindCluster function and the FindAllMarkers function were subsequently applied to identify distinct microglial clusters and their DEGs, respectively.

### DAM gene score

To elucidate the genetic overlaps between microglial subtypes and DAMs, we employed the DAM score metric. Specifically, we identified the genesets that were enriched in DAMs by referring to the work of Deczkowska and colleagues.^46^ Next, we translated these DAM genesets into their human homologs using the homologene package. Finally, we computed the DAM scores using the AddModuleScore function in the Seurat package.

### Construction of gene networks related to GO terms

In the initial step, we identified the DEGs for each cluster by utilizing the “FindAllMarkers()” function, configuring the parameters “only.pos = TRUE,” “min.pct = 0.25,” and “logfc.threshold = 0.25.” Subsequently, we selected the top 50 DEGs of these clusters based on the “avg_log_2_FC” values. Furthermore, we performed the GO enrichment analysis employing the “enrichGO()” function from the “clusterProfiler” package. The GO terms as well as the related genes are visualized using the “cytoscape” software.

### Supplementary information

Supplementary Information Figure S1

Supplementary Information Figure S2

Supplementary Information Figure S3

Supplementary Information Figure S4

Supplementary Information Figure S5

Supplementary Information Figure S6

Supplementary Information Figure S7

Supplementary Information Table S1

Supplementary Information Table S2

Supplementary Information Table S3

Supplementary Information Table S4

Supplementary Information Table S5

Supplementary Information Table S6

Supplementary Information Table S7

Supplementary Information Table S8

Supplementary Information Table S9

Supplementary Information Table S10

Supplementary Information Table S11

## Data Availability

Data accessibility is a crucial aspect of scientific research, and we have taken care to ensure that all relevant data from our study are accessible to the scientific community. Specifically, we have deposited our scRNA-seq datasets and spatial transcriptomic datasets (10× Visium and TFseqFISH) in the Gene Expression Omnibus (GEO) under the accession number GSE221692. Additionally, our supplementary materials contain a wealth of additional data that supports our conclusions.
