# Supplementary material for: Decoding the spatiotemporal regulation of transcription factors during human spinal cord development
Source: Cell Res. 2024 Jan 5;34(3):193–213. doi: 10.1038/s41422-023-00897-x (PMC10907391; doi:10.1038/s41422-023-00897-x)
Supplement: Supplementary file 1 — Supplementary Information Figure S1 [file 41422_2023_897_MOESM1_ESM.pdf]

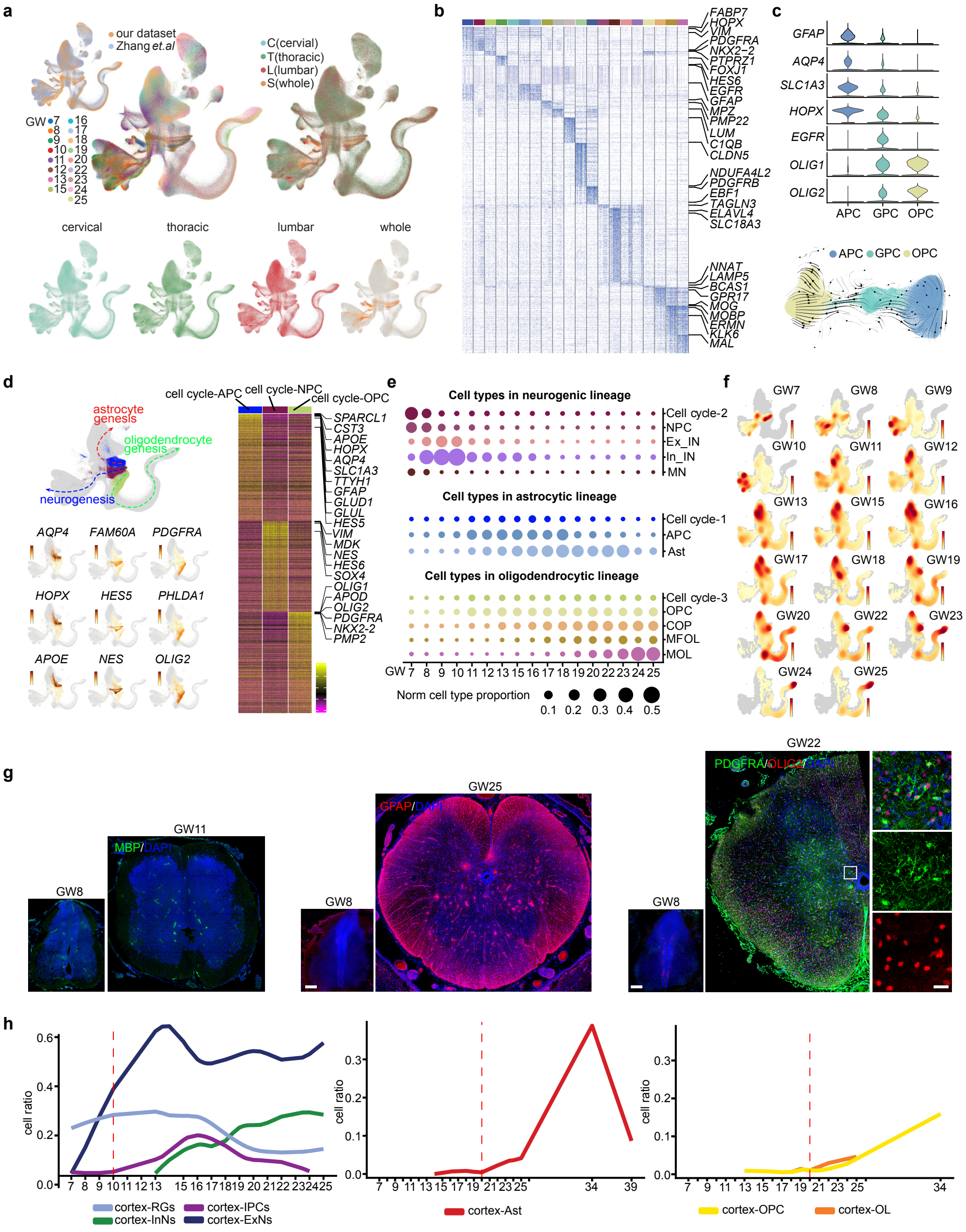

Fig. S1 A comprehensive transcriptomic repertoire of the developing human spinal cord. **a** The UMAP embedding exhibits color coding based on data sources (top right corner), gestational weeks (left), and spinal cord segments (right). The cells from different spinal cord segments or the entire spinal cord are visualized individually in UMAP (bottom). **b** A heatmap depicting the expression levels of differentially expressed genes across diverse cell types in developing human spinal cord is presented. **c** The upper part displays the shared and distinct gene expression

characteristics of APCs, OPCs, and GPCs. The lower part illustrates the inferred lineage relationships among APCs, GPCs, and OPCs using RNA velocity analysis. **d** The UMAP plot emphasizes cell cycle-1, 2, and 3 cells, which are associated with the lineages of astrocyte genesis, neurogenesis, and oligodendrocyte genesis, respectively. Meanwhile, the heatmap illustrates their distinctive gene expression patterns. Additionally, the expression profiles of some DEGs are visualized, with each dot representing an individual cell and colored according to the expression level (red, high; gray,low). **e** Dotplot illustrates the relative abundance of cell types within neuronal, astrocyte, and oligodendrocyte lineages across gestational weeks. **f** The distributions of cells at different gestational stages are displayed with a density map, in which high density is denoted by dark red. **g** The expression patterns of MBP, GFAP, PDGFRA, and OLIG2 in the developing human spinal cord tissues at various time points are visualized by performing immunostaining. **h** The changes in the cell ratio over gestational weeks in the neuronal, astrocytic, and oligodendrocytic lineages in the developing human brain are presented as fitted curves.
