# Supplementary material for: Decoding the spatiotemporal regulation of transcription factors during human spinal cord development
Source: Cell Res. 2024 Jan 5;34(3):193–213. doi: 10.1038/s41422-023-00897-x (PMC10907391; doi:10.1038/s41422-023-00897-x)
Supplement: Supplementary file 2 — Supplementary Information Figure S2 [file 41422_2023_897_MOESM2_ESM.pdf]

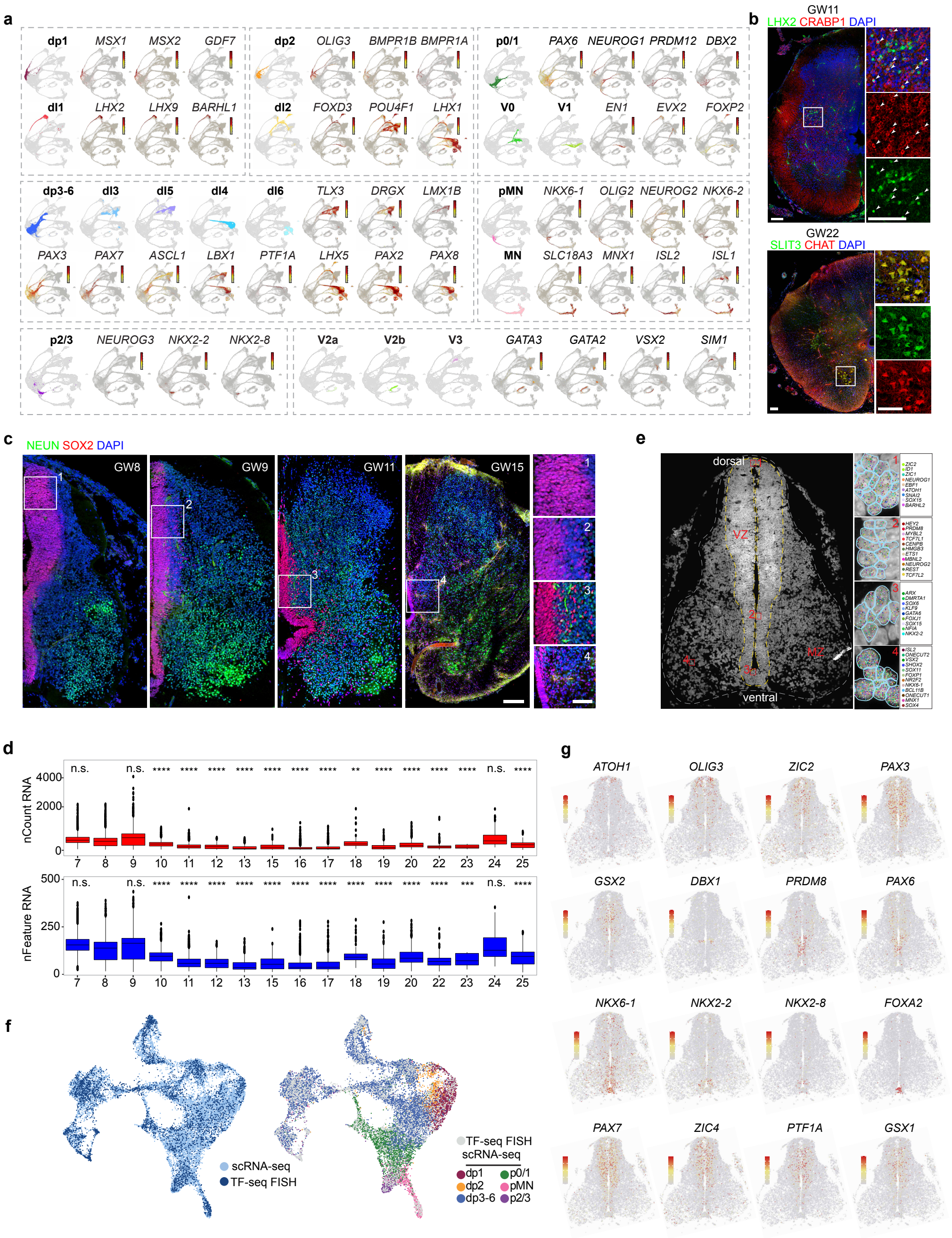

Fig. S2 Molecular and spatial characterization of distinct neural progenitor cells. a The expression profiles of well-known genes, used to classify diverse neural progenitors and neurons in distinct neuronal lineages, are depicted through a UMAP visualization. Each dot represents a single cell, colored based on expression level (red for high, gray for low). **b** Immunostaining results for LHX1 and CRABP1, as well as SLIT3 and CHAT, are presented. **c** Immunostaining results for

SOX2 and NeuN at different gestational stages are displayed. **d** The RNA levels in NPCs across distinct gestational stages are visualized by boxplot. The RNA levels at distinct stages are compared with that of GW8. n.s., not significant; ****, p value < 0.0001. **e** A coronal section of a human spinal cord at GW8 was subjected to TF-seqFISH and stained with DAPI. The VZ region, gray matter, and central canal were outlined by yellow dotted lines. Four areas of interest, denoted by red boxes, were magnified and visualized with each mRNA spot detected represented by a dot. **f** Visualization of integrated neural progenitor cell dataset from scRNA-seq and TF-seqFISH using UMAP. Each dot represents a single cell, color-coded according to its data source or NPC cell type. **g** The spatial expression profiles of transcription factors in a GW8 human spinal cord slice are presented, with each dot representing an individual cell colored according to the expression level (red for high, gray for low). The VZ is delineated by dotted lines for reference.
