# Supplementary material for: Decoding the spatiotemporal regulation of transcription factors during human spinal cord development
Source: Cell Res. 2024 Jan 5;34(3):193–213. doi: 10.1038/s41422-023-00897-x (PMC10907391; doi:10.1038/s41422-023-00897-x)
Supplement: Supplementary file 3 — Supplementary Information Figure S3 [file 41422_2023_897_MOESM3_ESM.pdf]

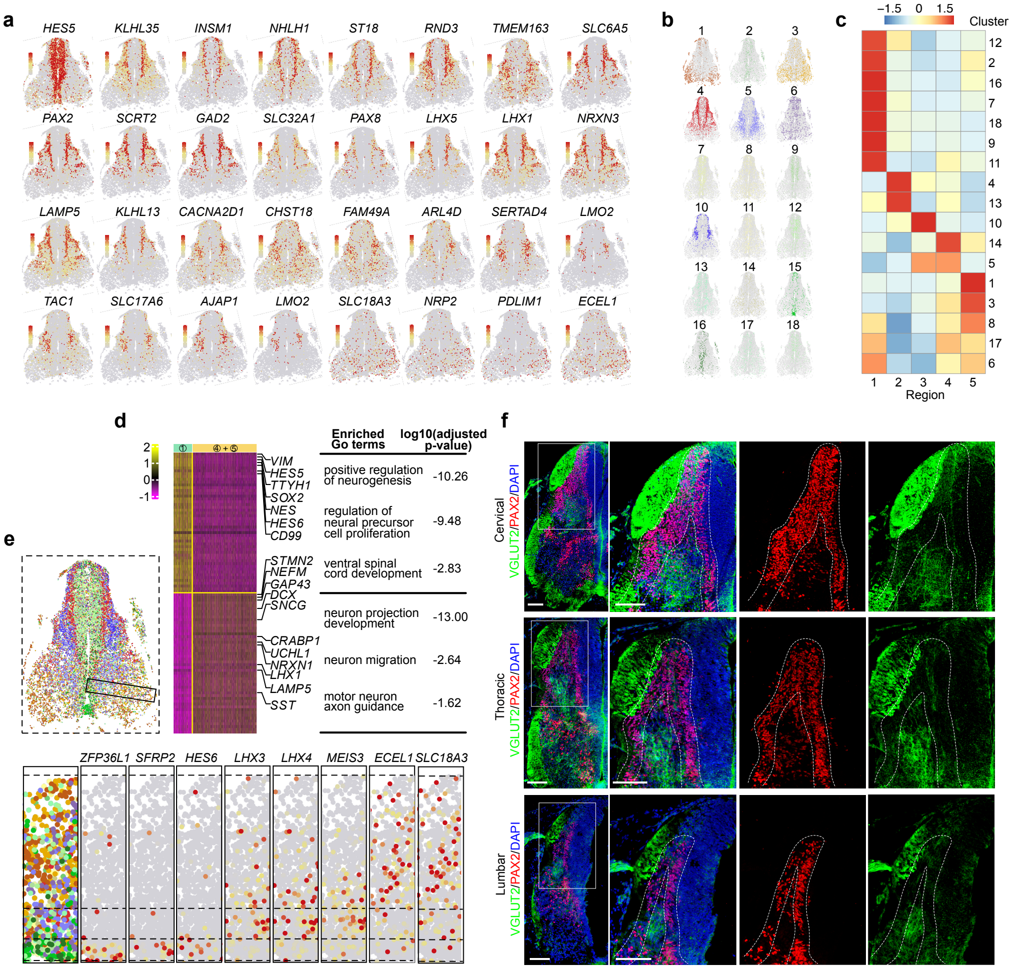

Fig. S3 Uncovering characteristic gene expression along the mediolateral axis of the developing human spinal cord. a Visualization of imputed gene expression profiles in developing human spinal cord with individual cells represented as dots and colored based on expression levels (red, high; gray, low). **b** The distribution of cells within the 18 unsupervised clusters is visualized. The cells from distinct clusters are colored differentially. **c** The correlation between the 18 unsupervised clusters and the 5 spatial domains is visualized by a heatmap. **d** A heatmap showcasing the expression profiles of DEGs between cells located in region 1 and regions 4 and 5 of the developing human spinal cord is presented herein, accompanied by the enriched gene ontology terms for the aforementioned DEGs. **e** Spatially-resolved laminar gene expression profiles along the medial-lateral axis are shown in this visualization. Each dot corresponds to an individual cell, color-coded based on its expression level (red, high; gray, low). The leftmost column provides a zoomed-in view of the boxed area. **f** The immunostaining for VGLUT2 and

PAX2 across distinct spinal segments are visualized to illustrate the presence of the sandwich-like neuronal structure across the entire developing human spinal cord.
