# Supplementary material for: Decoding the spatiotemporal regulation of transcription factors during human spinal cord development
Source: Cell Res. 2024 Jan 5;34(3):193–213. doi: 10.1038/s41422-023-00897-x (PMC10907391; doi:10.1038/s41422-023-00897-x)
Supplement: Supplementary file 4 — Supplementary Information Figure S4 [file 41422_2023_897_MOESM4_ESM.pdf]

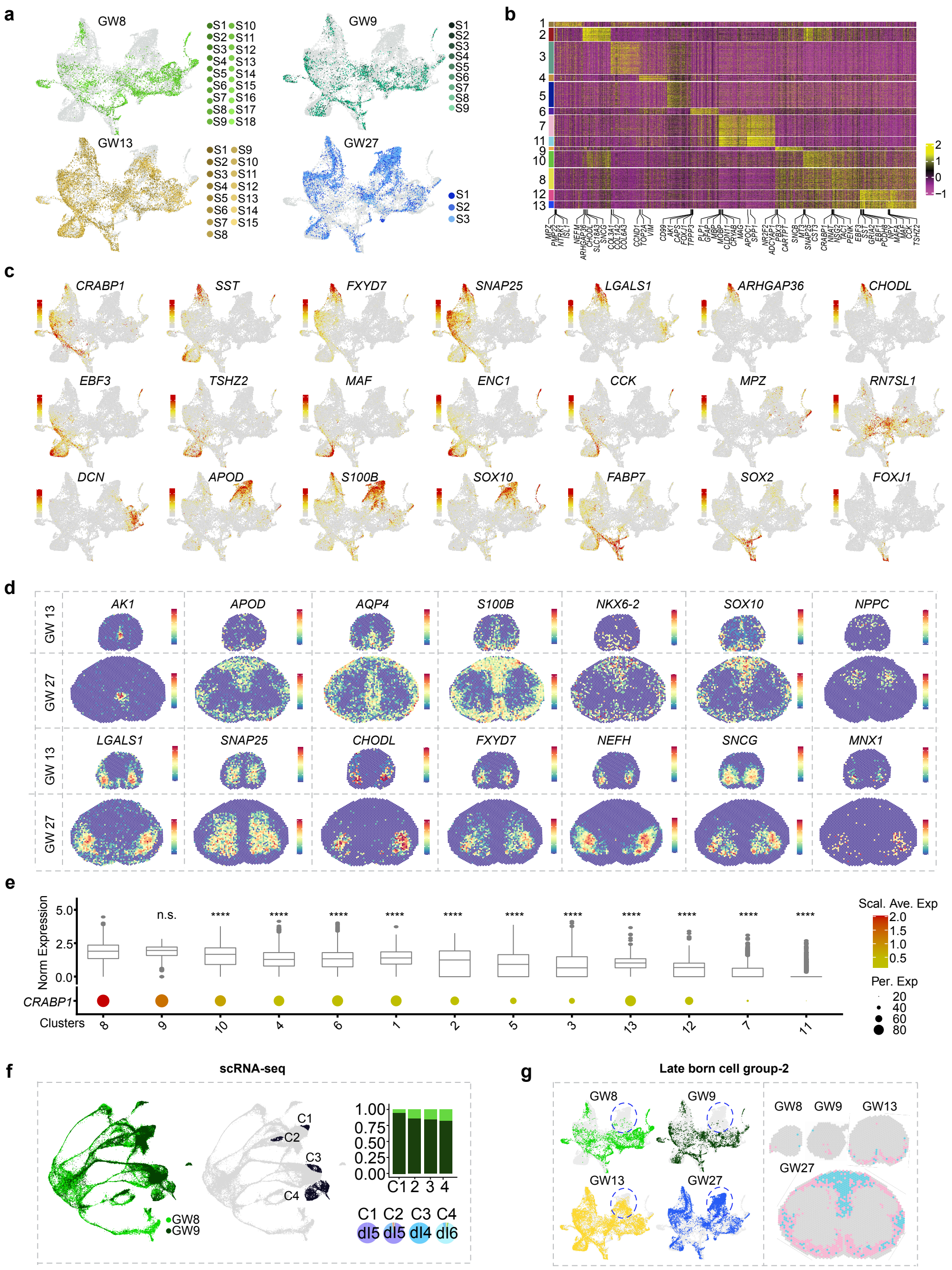

Fig. S4 Depicting the spatial characteristics of the developing human spinal cord using 10X Visium spatial transcriptomics. a The UMAP embedding is color-coded to show the individual slices analyzed at different gestational stages in the 10x Visium experiment, effectively

highlighting the reproducibility. **b** Heatmap depicting differential expression of genes among the cell types delineated in Fig. 4A. **c** The gene expression patterns are presented using UMAP visualization. Each dot on the UMAP represents an individual dot in the 10X Visium dataset, and its color is coded according to the expression level (red, high; gray, low). **d** The Visium dataset of GW13 and GW27 is used to visualize the spatially-resolved expression pattern of well-known genes utilized for cell type annotation. **e** The expression profiles of *CRABP1* across the distinct unsupervised clusters of the 10X Visium dataset are illustrated. The expression levels of *CRABP1* in these distinct clusters are compared with those in cluster 8. n.s., not significant, ****, p-value < 0.0001. **f** The delayed emergence of neurons exhibiting dI4, dI5, and dI6 identities is demonstrated in the scRNA-seq dataset, which includes cells from GW8 and GW9. Specifically, this dataset identified four cell groups with dI4, dI5, and dI6 identities in GW9. **g** Clusters 7 and 11 are not detected in early developmental stages at GW8 and GW9. The spatial distribution of these clusters is shown in the 10X Visium dataset of GW13 and GW27.
