# Supplementary material for: Decoding the spatiotemporal regulation of transcription factors during human spinal cord development
Source: Cell Res. 2024 Jan 5;34(3):193–213. doi: 10.1038/s41422-023-00897-x (PMC10907391; doi:10.1038/s41422-023-00897-x)
Supplement: Supplementary file 5 — Supplementary Information Figure S5 [file 41422_2023_897_MOESM5_ESM.pdf]

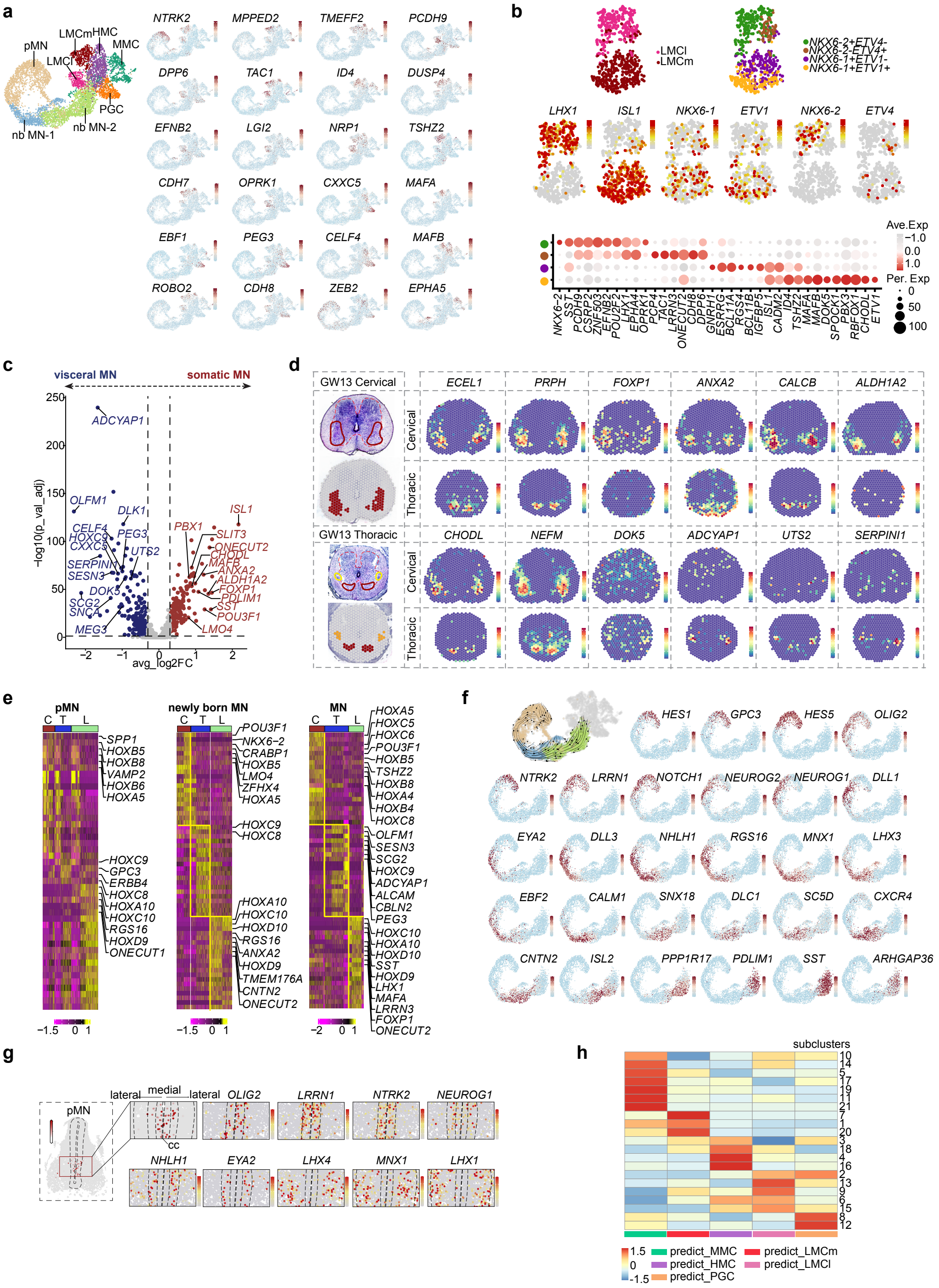

Fig. S5 The molecular and spatial explanation for the MN diversification. a The expression profiles of genes with specificity in distinct motor neuron columns are visualized using UMAP. Each dot represents an individual cell and is colored according to its expression level, with dark red indicating high expression and light blue indicating low expression. (dark red, high; light blue,

low). **b** Distinct subtypes of LMC cells with unique gene expression profiles and putative MN pool identities can be identified in the developing human spinal cord. **c** A volcano plot displaying differential gene expression between somatic and visceral motor neurons in the developing human spinal cord. **d** The spatial distribution of somatic and visceral MNs in the human spinal cord at GW13 is delineated by circles in HE staining images of cervical and thoracic slices and correspondingly highlighted in 10x Visium images. The region-specific gene expression in somatic or visceral MNs is also displayed in the 10x Visium dataset of cervical and thoracic slices of the developing human spinal cord at GW13. **e** Heatmap depicts differential gene expression in cervical, thoracic, and lumbar subgroups of motor neurons, newly born motor neurons, and progenitor motor neurons in the developing human spinal cord. **f** The developmental trajectory from pMN to newly born MN-2 is inferred by RNA velocity analysis, and the expression profiles of genes along this continuum are visualized using UMAP. Each dot represents an individual cell and is colored based on the expression level (dark red, high; light blue, low). **g** The spatial expression pattern of genes that regulate pMN differentiation is inferred from the TF-seqFISH dataset. **h** The heatmap visualizes the correlation between the unsupervised subclusters of newly born motor neurons and their predicted column identities.
