# Supplementary material for: Decoding the spatiotemporal regulation of transcription factors during human spinal cord development
Source: Cell Res. 2024 Jan 5;34(3):193–213. doi: 10.1038/s41422-023-00897-x (PMC10907391; doi:10.1038/s41422-023-00897-x)
Supplement: Supplementary file 6 — Supplementary Information Figure S6 [file 41422_2023_897_MOESM6_ESM.pdf]

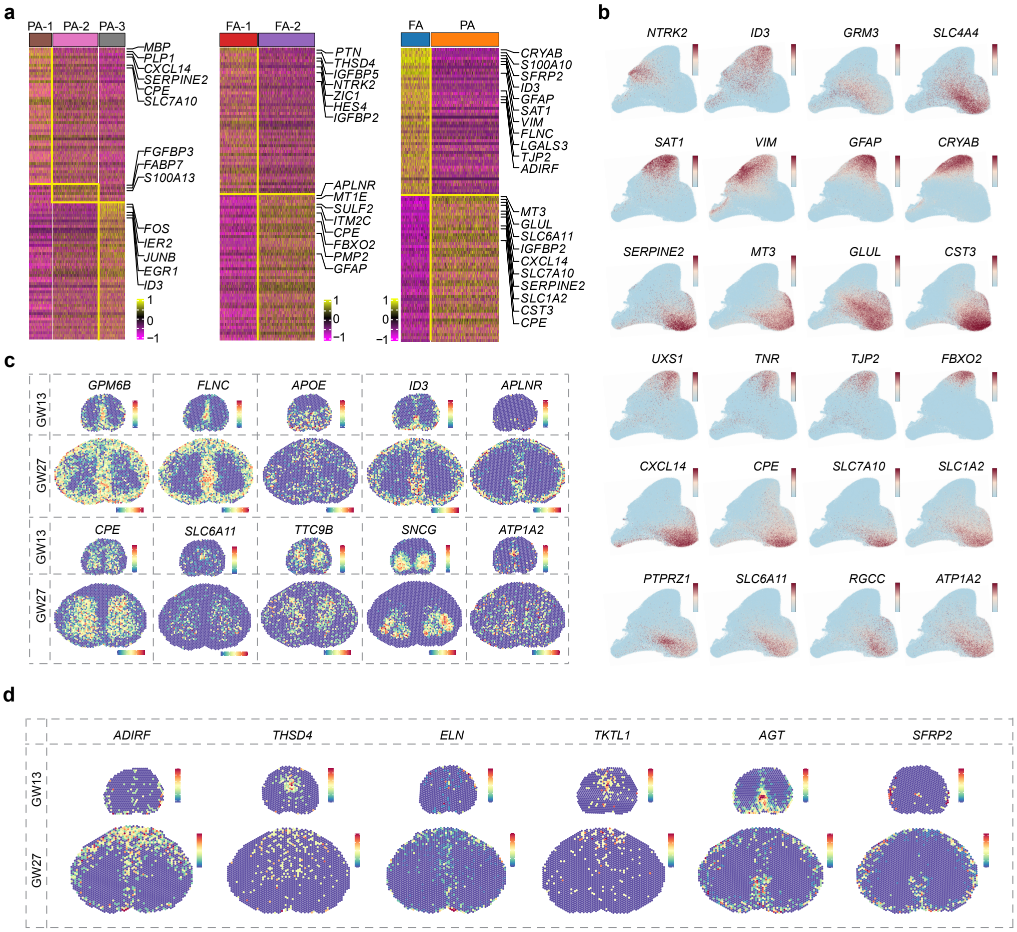

Fig. S6 The molecular and spatial heterogeneity of astrocytes in the developing human spinal cord. a A heatmap showcasing the distinct gene expression profiles between PA-1, PA-2, and PA-3 (left), FA-1 and FA-2 (middle), and FA and PA (right). **b** Distinctive gene expression is represented by a UMAP visualization. Each dot denotes a single cell, colored based on its expression level (dark red, high expression; light blue, low expression). **c** The spatial expression profiles of FA specific and PA specific genes are delineated using the 10X Visium platform. **d** Visualization of the spatially resolved gene expression profiles with dorsal and ventral preferences in 10x Visium Dataset.
