# Supplementary material for: Decoding the spatiotemporal regulation of transcription factors during human spinal cord development
Source: Cell Res. 2024 Jan 5;34(3):193–213. doi: 10.1038/s41422-023-00897-x (PMC10907391; doi:10.1038/s41422-023-00897-x)
Supplement: Supplementary file 7 — Supplementary Information Figure S7 [file 41422_2023_897_MOESM7_ESM.pdf]

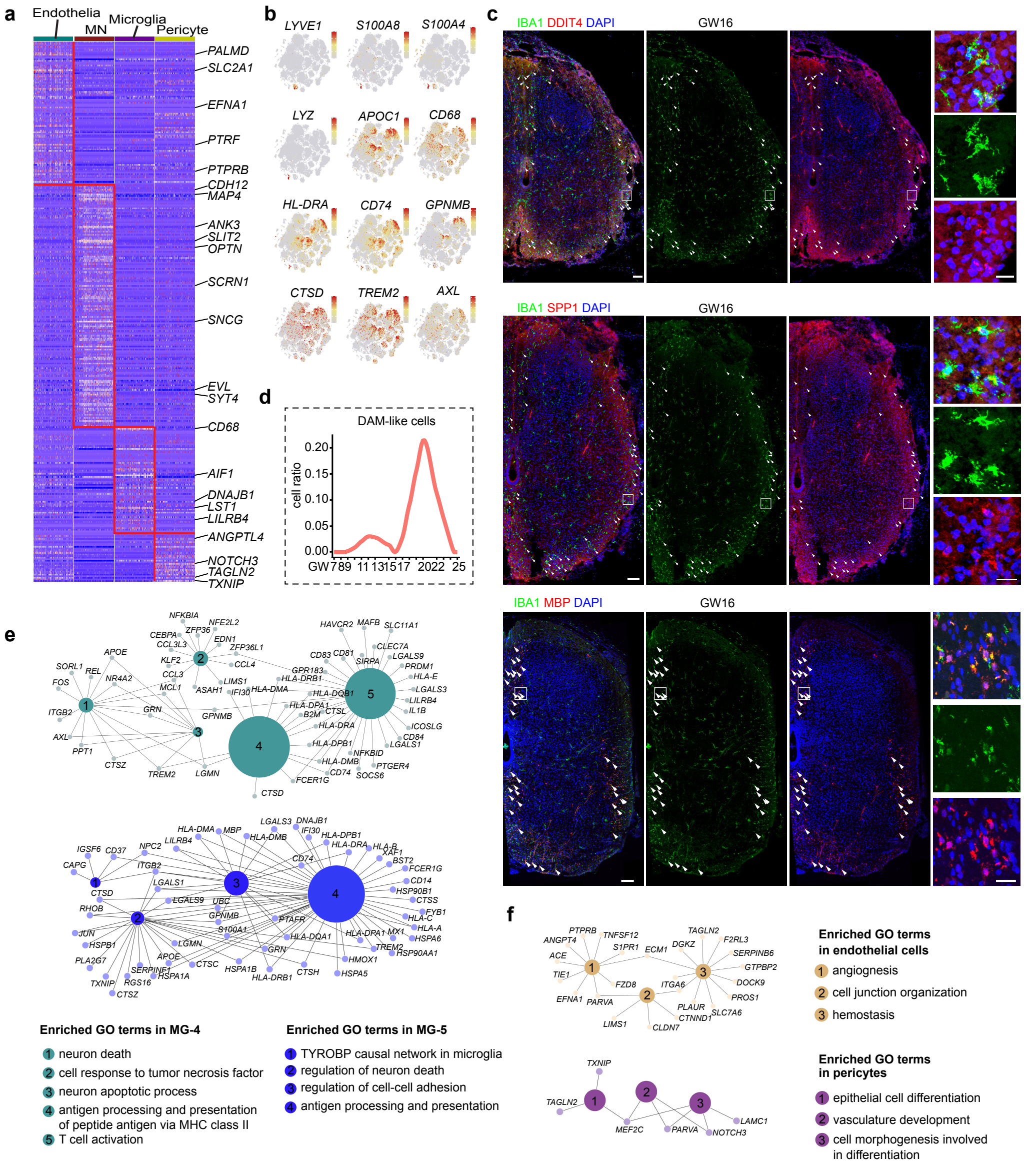

Fig. S7 Illuminating cell type-specific regulation of ALS pathogenesis. a A heatmap showing the expression profiles of ALS risk genes probed by GWAS that are enriched in distinct cell types. **b** The expression profiles of myeloid cell hallmark genes and DAM-related genes is visualized. Each dot represents a single cell and is colored according to the expression level (gray for low, red for high). **c** The immunostaining results depicting IBA1 and DDI4, IBA1 and SPP1 as well as, IBA1 and MBP are presented. The white arrows indicate the double-positive cells expressing both IBA1 and DDIT4, IBA1 and SPP1 or IBA1 and MBP. **d** The dynamic of cell ratio of DAM-like microglia is analyzed based on the scRNA-seq data. **e** The enriched GO terms and the

corresponding genetic networks for MG-4 and MG-5 have been analyzed based on their DEGs. **f** The enriched GO terms and corresponding genetic networks for endothelial cells and pericytes have been analyzed based on their DEGs.
